# Porcine gut microbiota in mediating host metabolic adaptation to cold stress

**DOI:** 10.1038/s41522-022-00283-2

**Published:** 2022-04-05

**Authors:** Yu Zhang, Lan Sun, Run Zhu, Shiyu Zhang, Shuo Liu, Yan Wang, Yinbao Wu, Sicheng Xing, Xindi Liao, Jiandui Mi

**Affiliations:** 1 Guangdong Laboratory for Lingnan Modern Agriculture, South China Agricultural University, Guangzhou, 510642 China; 2 National Engineering Research Center for Breeding Swine Industry, College of Animal Science, South China Agricultural University, Guangzhou, 510642 China; 3 Guangdong Provincial Key Lab of Agro-Animal Genomics and Molecular Breeding and Key Lab of Chicken Genetics, Breeding and Reproduction, Ministry of Agriculture, Guangzhou, 510642 China

**Keywords:** Metagenomics, Metagenomics

## Abstract

The gut microbiota plays a key role in host metabolic ﻿thermogenesis by activating UCP1 and increasing the browning process of white adipose tissue (WAT), especially in cold environments. However, the crosstalk between the gut microbiota and the host, which lacks functional UCP1, making them susceptible to cold stress, has rarely been illustrated. We used male piglets as a model to evaluate the host response to cold stress via the gut microbiota (four groups: room temperature group, *n* = 5; cold stress group, *n* = 5; cold stress group with antibiotics, *n* = 5; room temperature group with antibiotics, *n* = 3). We found that host thermogenesis and insulin resistance increased the levels of serum metabolites such as glycocholic acid (GCA) and glycochenodeoxycholate acid (GCDCA) and altered the compositions and functions of the cecal microbiota under cold stress. The gut microbiota was characterized by increased levels of *Ruminococcaceae*, *Prevotellaceae*, and *Muribaculaceae* under cold stress. We found that piglets subjected to cold stress had increased expression of genes related to bile acid and short-chain fatty acid (SCFA) metabolism in their liver and fat lipolysis genes in their fat. In addition, the fat lipolysis genes CLPS, PNLIPRP1, CPT1B, and UCP3 were significantly increased in the fat of piglets under cold stress. However, the use of antibiotics showed a weakened or strengthened cold tolerance phenotype, indicating that the gut microbiota plays important role in host thermogenesis. Our results demonstrate that the gut microbiota-blood-liver and fat axis may regulate thermogenesis during cold acclimation in piglets.

## Introduction

The intestinal microbial community is a complex ecosystem composed of various microorganisms with which the host interacts from birth^1^. The host provides a habitat for specific intestinal microbial communities, which in turn provide nutrition and energy for the host and promote the acquisition of food energy and the storage of white fat^2–4^. Changes in ambient temperature are among the strongest physiological stimuli that increase the formation and activity of thermogenic fat^5,6^. In mammals, nonshivering thermogenesis (NST) generates metabolic heat to maintain body temperature in response to cold^7^. When the temperature drops, the sympathetic nervous system releases β-adrenergic agonists, such as norepinephrine (NE), which stimulates β-adrenergic receptors on brown adipocytes and initiates a series of downstream events, including the transcription of UCP1^8^. Brown adipose tissue (BAT), which is dependent on the UCP1 process, undergoes uncoupling of the mitochondrial electron transport chain to produce heat from the oxidation of carbohydrates and lipids^9^. However, the activation of metabolic heat production in response to cold is not restricted to BAT. The BAT-like phenotype is derived from WAT depots, and brown adipocytes are formed under exposure to cold in a process known as “browning”^8^. Intestinal microbes in mice regulate fat accumulation by promoting thermogenesis^10^. Cold exposure led to significant changes in the composition of intestinal microbes, referred to as the cold microbiota, and the relative abundance of the Firmicutes phylum increased and that of Bacteroidetes decreased in feces under cold stress compared with room temperature (RT). Interestingly, transplantation of the “cold microbiota” increased the browning of white fat and insulin sensitivity in recipient mice, which indicated that the “cold microbiota”-transplanted mice had resistance to cold, which was partly mediated by the browning of white fat^11^. Further exploration of the role of the intestinal microbiota as a cold-induced heat-producing system showed that cold exposure changed the cecal microbes of Brandt’s voles (*Lasiopodomys brandtii*) and increased the concentration of short-chain fatty acids (SCFAs). In addition, injection of NE also resulted in long-term changes in the intestinal microbial structure, and it was confirmed that transplantation of the “cold microbiota” increased thermogenesis through activation of cAMP-PKA-pCREB signaling, which might have been due to the interaction of the intestinal microbiota with host neurotransmitters and the joint regulation of heat production and energy consumption in the body under exposure to cold^12^. Generally, intestinal microbes adapt to changes in the external temperature by adjusting the browning of white fat and the activity of brown fat^10,11,13–15^.

Changes in warm environments also play a vital role in the health of piglets^16^. Since the thermoregulatory mechanism of piglets has not been fully developed, the ambient temperature required for piglets’ survival is high. Although thermal insulation measures are usually undertaken during piglet breeding, cold stress syndrome is still very common in piglets in actual production. Moreover, most breeds of pigs have no external brown fat reservoir, only a white fat reservoir. Many pigs have lost the NST effect of UCP1-mediated brown fat decomposition and rely mainly on shivering thermogenesis^17^. The lack of a functional UCP1 gene, which is very important for thermogenesis, leads to a decreased ability to regulate body temperature and to an increase in cold sensitivity under cold stress. In addition, in piglet, cold stress causes decreased resistance, induces intestinal damage and microecological imbalance, causes diarrhea and various other diseases, and reduces the growth rate, leading to piglet death^18,19^. Intestinal microbes may relieve cold stress by providing 15–20% of the energy to the host, severely affecting the production efficiency of pigs. Therefore, maintaining the intestinal health of piglets and reducing cold stress in the environment play important roles in the health of piglets, so it is particularly important to alleviate cold stress in piglets.

Therefore, the purpose of this study was to evaluate the responses of piglet intestinal microbes under cold stress intervention and to preliminarily explore the possible mechanisms by which intestinal microbes mediate cold stress in piglets to provide a theoretical basis and technical support for improving cold resistance and maintaining the intestinal health of piglets.

## Results

### Cold exposure changes blood metabolism parameters in piglets

**Fig. 1:**
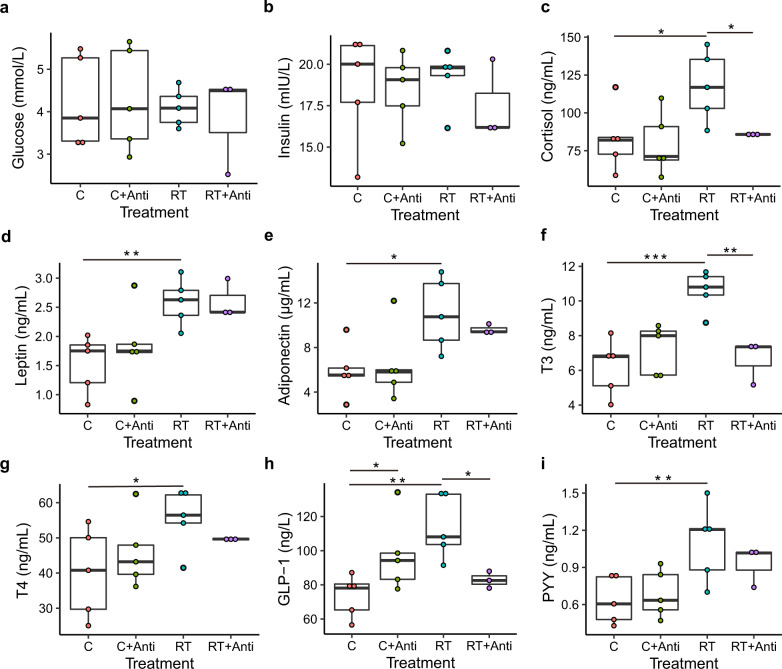
Effect of cold stress on the serum characteristics of piglets. **a** Glucose level in serum. **b** Insulin level in serum. **c** Cortisol level in serum. **d** Leptin level in serum. **e** Adiponectin level in serum. **f** Triiodothyronine (T3) level in serum. **g** Thyroxine level in serum. **h** Glucagon-like peptide-1 (GLP-1) level in serum. **i** Peptide YY (PYY) level in serum. C, C + Anti, RT, and RT + Anti represent the cold stress, cold stress with antibiotics, room temperature, and room temperature with antibiotics groups, respectively. The data are the means ± SEMs (*n* = 3 or 5 per group). Statistical significance was determined using Wilcoxon test (*p < 0.05, **p < 0.01, ***p < 0.001).

To explore the effects of cold exposure on the metabolism of piglets, we tested the energy metabolism parameters of blood serum after 48 h of cold exposure with or without the addition of broad-range antibiotics to drinking water to deplete the intestinal microbiota (Supplementary Data 1). Glucose and insulin concentrations did not differ between the groups (Fig. 1a, b). The cortisol concentration was lower under cold stress than under RT (Fig. 1c, *P* < 0.05). The leptin and adiponectin concentrations were lower under cold stress than under RT regardless of antibiotic use (Fig. 1d, e, *P* < 0.05). However, under the same conditions, depletion of the gut microbiota did not influence the leptin and adiponectin concentrations in blood serum (*P* > 0.05). The T3 concentration was decreased by cold exposure compared with that at RT (Fig. 1f, *P* < 0.05). After depleting the piglet intestinal microbiota with antibiotics, the T3 concentration was significantly lower than that under RT alone, which indicated that the intestinal microbiota played an important role in maintaining T3 homeostasis (Fig. 1f, *P* < 0.05). In response to cold exposure, T4 yielded the same result as T3 without antibiotic use; however, there was no difference under RT with or without antibiotic addition (Fig. 1g). A tendency bordering on significance toward decreased GLP-1 concentrations were observed in the blood serum of cold-exposed or RT + antibiotic-treated piglets (Fig. 1h). The PYY concentration had the same trend as the GLP-1 response to cold exposure; however, antibiotic use did not result in a significant difference (Fig. 1i). These data further suggested that cold exposure selectively affected blood energy metabolism parameters and that most of the parameters were decreased.

### Cold exposure increases IgA levels and insulin sensitivity

**Fig. 2:**
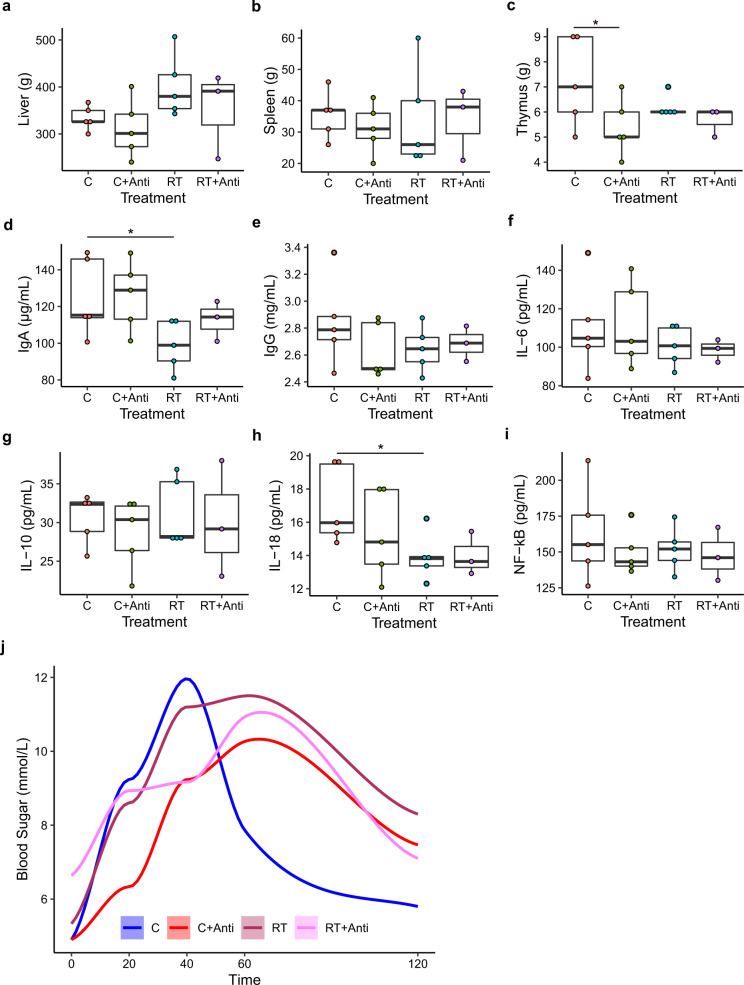
Effects of cold stress on the weight of organs, immune factors, and cytokines in serum and glucose tolerance tests. **a** Liver weight. **b** Spleen weight. **c** Thymus weight. **d** IgA level in serum. **e** IgG level in serum. **f** IL-6 level in serum. **g** IL-10 level in serum. **h** IL-18 level in serum. **i** NF−kB level in serum. **j** Glucose tolerance test. The data are the means ± SEMs (*n* = 3 or 5 per group). Statistical significance was determined using Wilcoxon test (*p < 0.05, **p < 0.01, ***p < 0.001).

To investigate whether cold exposure could influence the weight of important organs, we measured the liver, spleen, and thymus weights after slaughter (Fig. 2a–c and Supplementary Data 2). Only liver weight showed a decreasing trend under cold stress compared with that at RT in the nonantibiotic group (Fig. 2a, *P* = 0.059). The IgA and IL-18 concentrations in the blood serum of piglets were increased under cold stress, while there were no significant differences under the other conditions, both with and without antibiotic use (Fig. 2d, h and Supplementary Data 3). The IgG, IL-6, IL-10, and NF-kB concentrations did not significantly differ among the four groups (Fig. 2e–g, i and Supplementary Data 3). We also determined glucose tolerance by measuring the blood sugar concentration at different times after oral administration of glucose (Fig. 2j). The results showed that cold-exposed piglets without antibiotic use had an elevated glucose peak following glucose gavage compared with other groups after 40 min (Fig. 2j), but also exhibited the fastest clearance. Interestingly, nearly the same initial glucose peak was observed between the cold and cold+antibiotics groups, which suggested that oral glucose was rapidly taken up in cold-exposed piglets without antibiotic use. However, piglets subjected to intestinal microbiota depletion at RT or under cold stress showed delayed peak appearance at 60 min, and the speed of clearance was slower than that under cold stress without antibiotics but higher than that at RT without antibiotics. In our models, the duodenal mucosal thickness, villus length, crypt depth, and cecal mucosal thickness increased in the cold-exposed piglets compared with RT-treated piglets (Supplementary Data 3). Interestingly, there was an antibiotic-dependent effect on the above parameters, which was enhanced upon intestinal microbiota depletion at RT. However, under cold stress with antibiotic treatment, the duodenal mucosal thickness and villus length decreased compared with those under cold stress without antibiotics (Supplementary Fig. 3a, b) but the duodenal crypt depth increased.

### Cold exposure changes the microbiota composition of the contents and epithelial surface of the cecum

**Fig. 3:**
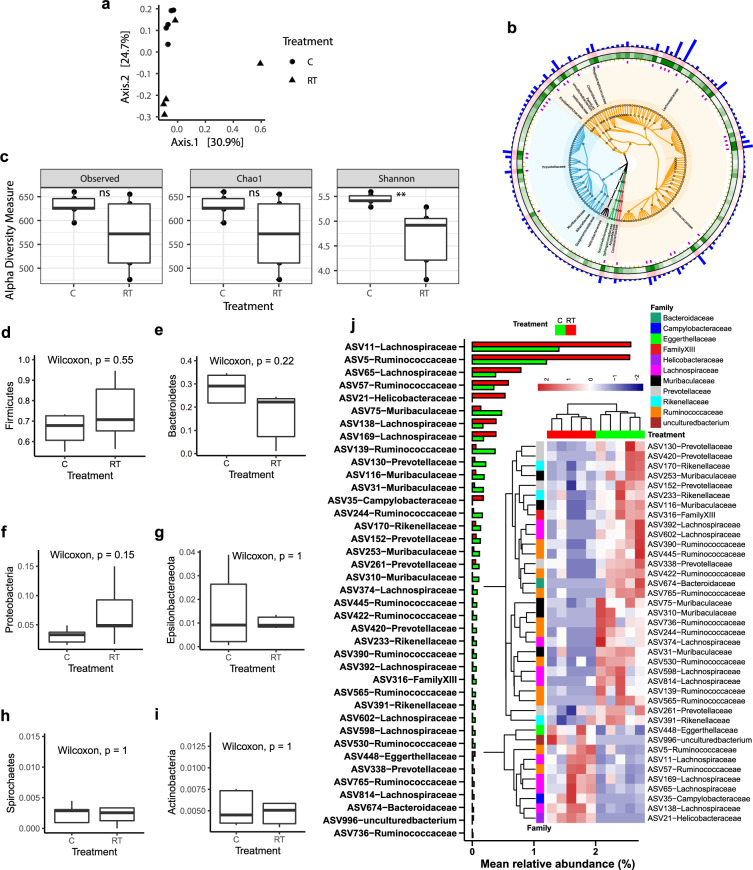
Cold exposure changes the cecal microbiota composition. **a** Principal coordinates analysis (PCoA) based on the weighted UniFrac distances. Each symbol represents a single sample of the cecal content after 48 h of cold exposure (*n* = 5) or RT controls (*n* = 5 per group). **b** Heatmap tree comparing the most abundant ASVs from the cecum content of 48 h cold-exposed animals (*n* = 5, inner green rings) and RT controls (*n* = 5, outer pink rings) and their phylogenetic relationships. The bar represents abundant ASVs. Firmicutes and Bacteroidetes appear in orange and blue colors, respectively. **c** The alpha diversity of ASVs. **d**–**i** Boxplot of the microbiota of the cecal contents at the phylum level. **j** The different ASVs with a bar and heatmap with *P* < 0.05. The data are the means ± SEMs (*n* = 5 per group).

To investigate whether cold stress causes changes in the intestinal microbiota, we collected cecal content and epithelium-attached samples from cold-exposed and RT-treated piglets (Fig. 3 and Supplementary Fig. 4). Determination of the composition by 16S rRNA marker gene sequencing, followed by principal coordinates analysis (PCoA) based on the weighted UniFrac distance, revealed large alterations in the microbiota compositions under cold stress (Fig. 3a, PERMANOVA test *p-*value = 0.037). The abundant phyla were *Firmicutes*, *Bacteroidetes*, *Proteobacteria*, *Epsilonbacteraeota*, and *Actinobacteria*. Firmicutes was the most abundant phylum in all the samples (on average 72.16%), followed by Bacteroidetes (on average 23.13%) (Fig. 3b). At the family level, Lachnospiraceae, Ruminococcaceae, and Prevotellaceae were the most abundant families in all the samples, with proportions of 36.95, 22.77, and 20.43%, respectively, on average. The alpha diversity increased after cold exposure, but only the Shannon index was significantly higher than that under RT (Fig. 3c). However, no significant differences were found between the cold and RT treatments at the phylum level (Fig. 3d–j). We further investigated the differences in ASVs using STAMP software and FDR-adjusted *p*-values. We found 39 ASVs with significantly different relative abundances between the cold and RT treatments, most of which (20 ASVs) showed higher abundances under cold stress than under RT (Fig. 3k, *P* < 0.05). These 20 ASVs were distributed in the *Lachnospiraceae*, *Ruminococcaceae*, *Prevotellaceae, Rikenellaceae,* and *Muribaculaceae* families. The abundance of total of 10 ASVs was elevated at RT compared with cold stress, and these ASVs belonged to the *Eggerthellaceae, Campylobacteraceae*, and *Helicobacteraceae* families (Fig. 3j, *P* < 0.05).

The intestinal epithelial surface, where key signal transporters are located, such as SCFA receptors, is the most important site for interactions between the intestinal microbiota and the host. Thus, we also examined the cecal epithelium-attached microbiota using 16S rRNA sequencing (Supplementary Fig. 4). PCoA showed little overlap between samples of the gut microbiota under cold stress and at RT (Supplementary Fig. 4a, PERMANOVA test *p* value = 0.425). All the alpha diversity values also showed an increasing trend for the cold-exposed piglets, but no significant difference was observed (Supplementary Fig. 4c). The most abundant phyla in the intestinal epithelium-attached microbiota were the same as those in the cecum. However, the order of the relative abundances of these phyla was different from that in the cecum and included, with averages of 50.02, 22.44, 17.29, 8.73, and 1.00%, respectively. The most abundant families were *Prevotellaceae, Lachnospiraceae, Ruminococcaceae, and Muribaculaceae*, with relative abundance of 44.29, 9.81, 6.55, and 2.18%, respectively. The phyla Firmicutes and Spirochaetes showed an increasing trend compared with their levels at RT (Supplementary Fig. 4f and S4i, *P* = 0.071, and 0.056, respectively). The other phyla were not influenced under cold stress (Supplementary Fig. 4d, g, h, j). At the ASV level, the relative abundances of 14 ASVs were significantly different between the two groups, with 12 showing higher abundances under cold stress than at RT (Supplementary Fig. 4e, *P* < 0.05). A total of 12 ASVs belonged to *Prevotella*, *Alloprevotella, Ruminococcaceae, Muribaculaceae*, and *Erysipelotrichaceae* (Supplementary Fig. 4e). We also calculated the differences in the relative abundances of the bins from the metagenomic sequencing analysis (Supplementary Figs. 5, S6). The relative abundances of a total of 7 bins were found to be significantly different between the cold and RT conditions in the contents and on the epithelial surface of the cecum. The relative abundances of *Ruminococcaceae*, *Prevotellaceae*, and *Muribaculaceae* were higher in both the contents and on the epithelial surface of the cecum (Supplementary Fig. 6).

### Cold exposure changes the functions of the microbiota in the cecum

**Fig. 4:**
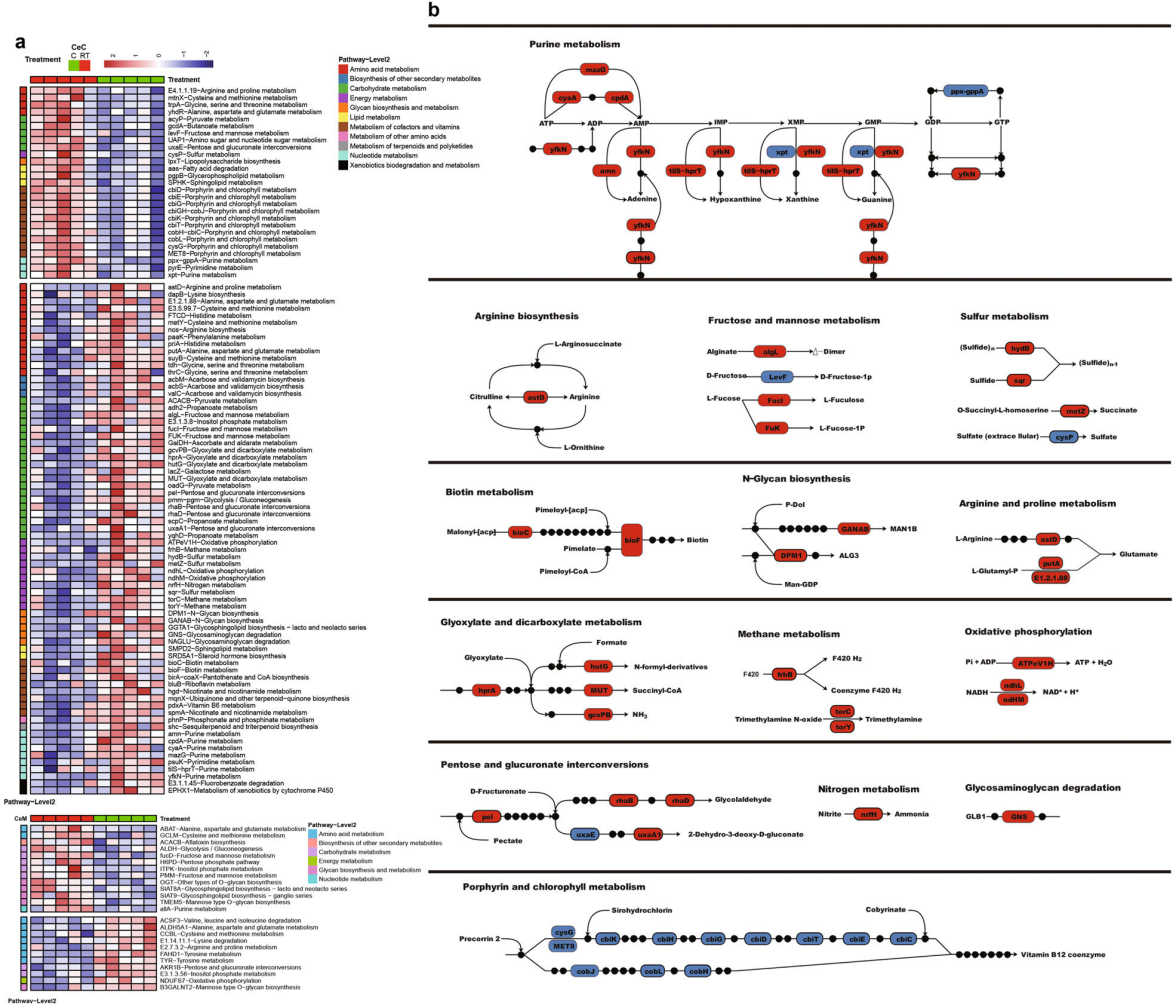
Cold exposure changes the function of microbiota genes involved in metabolism in the cecum, as indicated by the KO genes and KEGG pathway modules. **a** Heatmap differences at the metabolic level of the microbiota of the contents and epithelial surface of the cecum. **b** Representative pathway with the highest difference in abundance. Red indicates higher expression of genes, and blue indicates lower expression of genes, in piglets under cold stress compared with RT. CeC and CeM represent the cecal content and the cecal epithelial surface respectively. The data are the means ± SEMs (*n* = 5 per group).

The functions of the gut microbiome were confirmed using metagenomics sequencing. A total of 99 Kyoto Encyclopedia of Genes and Genomes (KEGG) orthologous (KO) genes in the metabolic catalog were significantly different between the cold and RT conditions (*P* < 0.05), 72 of which were more highly expressed in the cecal microbiota under cold stress than at RT (Fig. 4a). Changes in gene abundances are shown by pathway representation (Fig. 4b). Among the most differentially abundant pathways, eight genes in purine metabolism, one gene in arginine biosynthesis, four genes in fructose and mannose metabolism, four genes in sulfur metabolism, two genes in biotin metabolism, two genes in N-glycan biosynthesis, three genes in arginine and proline metabolism, four genes in glyoxylate and dicarboxylate metabolism, three genes in methane metabolism, three genes in oxidative phosphorylation, five genes in pentose and glucuronate interconversions, one gene in nitrogen metabolism, and one gene in glycosaminoglycan degradation were found to be associated with cold exposure (Fig. 4b). Increased production of purines, including adenine, hypoxanthine, xanthine, and guanine, might be associated with higher energy requirements for microbiome growth and metabolism. Gas production in the intestine is important for energy balance and even for signal transport. Hydrogen, and trimethylamine for methane metabolism, ammonia for nitrogen, glyoxylate and dicarboxylate metabolism, and sulfide and succinate for sulfur metabolism were elevated under cold stress. However, 12 genes involved in porphyrin and chlorophyll metabolism were found to be significantly associated with RT conditions, and were responsible for producing vitamin B12 coenzymes (Fig. 4b). On the epithelial surface of the cecum, a total of only 24 KO genes were significantly different under the metabolism category. Eleven of these genes were elevated under cold stress compared with RT and were mainly (7/11) associated with amino acid metabolism (Fig. 4a). Nine of 13 KO genes were more highly expressed at RT and related to carbohydrate metabolism and glycan biosynthesis and metabolism.

A total of 140 other KO genes in the cecum were found to be significantly differentially abundant between the two groups (*P* < 0.05) (Supplementary Fig. 7). In contrast, the levels of 56/140 KO genes were elevated at RT, and 84/140 KO genes were dominant under cold stress (Supplementary Fig. 7a). The functions adherence, spore germination, and motility belonging to protein families: signaling and cellular process pathways, were elevated under RT compared with cold stress, which indicated that the microbiome had greater capacity to become established or move to the proper microenvironment. In protein families: genetic information processing, 16/35 functions were elevated at RT, and 19/35 functions were elevated under cold stress. The functions of the cecal epithelium are shown in Supplementary Fig. 7b. These differences might be attributed not only to the microbiome but also to the inclusion of the host.

### Cold exposure elevates amino acid and bile acid metabolism levels

**Fig. 5:**
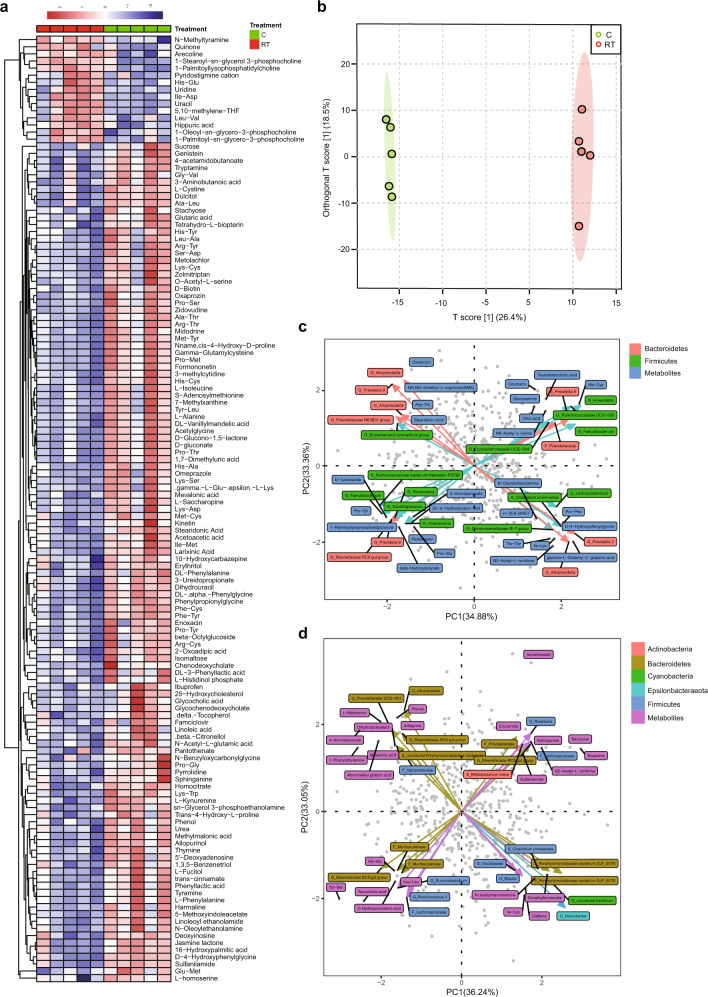
Different metabolomic alterations in serum in the positive model and strong correlation with the microbiota of the contents and epithelial surface of the cecum. **a** Heatmap of differences in serum metabolites under cold stress. **b** Partial least-squares discriminant analysis (PLS-DA) of metabolites in serum. **c** Correlation of metabolite in serum with the microbiome of the cecal content. **d** Correlation of metabolites with the microbiota of the epithelial surface of the cecum. The data are the means ± SEMs (*n* = 5 per group).

To identify the metabolites were related to cold exposure in the host, we detected blood metabolites using metabolomics. There were 133 significant metabolites in the positive model between the two groups (Fig. 5a), 120 of which were dominant under cold stress. The OPLS score also showed that the two groups were significantly separated (Fig. 5b). We further used targeted metabolomics to confirm the results for bile acid in serum and VFAs in the cecal content (Supplementary Fig. 8). We also used the microbe-metabolite vector (mmvec) to confirm the correlation between the microbiota and metabolites according to a previous report^20^. After analysis, we found that the levels of stearidonic acid, Arg-Thr, oxaprozin, glucosamine, His-Cys, Pro-Tyr, N-oleoylethanolamine, D-4-hydroxyphenylglycine, and glutamic acid were higher under cold stress than under RT, and a higher correlation was observed between metabolites and the microbiome in the cecum (Fig. 5a, c). Stearidonic acid was positively correlated with the *Prevotellaceae* NK3B31 group, *Ruminantium* group, *Alloprevotella*, and *Prevotella* 9 (Fig. 5c). Oxaprozin was most highly correlated with *Prevotella* 9 and *Alloprevotella*. Glucosamine and His-Cys were correlated with *Prevotella* 9 and *Ruminococcaceae* UCG-005. N-oleoylethanolamine was correlated with the *Christensenellaceae* R-7 group and *Alloprevotella*. D-4-hydroxyphenylglycine was correlated with *Prevotella 2*. Phenol, His-Ala, sphinganine, and sulfanilamide were more abundant among the blood metabolites and had a high correlation with the microbiome of the mucosal surfaces of the cecum (Fig. 5a, d). Phenol was correlated with *Alloprevotella* and *Prevotellaceae* UCG-001. His-Ala was correlated with *Muribaculaceae*. Sphinganine and sulfanilamide were both correlated with the *Prevotellaceae* and *Rikenellaceae* RC9 gut groups.

There were 67 significant metabolites in the negative model between the two groups (Supplementary Fig. 9a, b), 28 of which were elevated under cold stress. However, only a few metabolites, including 2-dehydro-3-deoxy-D-gluconate, anthranilic acid (vitamin L1), and 3-hexanone, were found to be enriched under cold stress and to have a strong correlation with the microbiome. 2-Dehydro-3-deoxy-D-gluconate was strongly correlated with *Prevotella* 9 in the cecum (Supplementary Fig. 9c), anthranilic acid (vitamin L1) was correlated with *Prevotellaceae* UCG-003 on the mucosal surfaces of the cecum (Supplementary Fig. 9d), and 3-hexanone was correlated with *Parabacteroides* and *Prevotellaceae* UCG-003 on the mucosal surfaces of the cecum.

### Cold stress increases the expression of UCP3 in fat

**Fig. 6:**
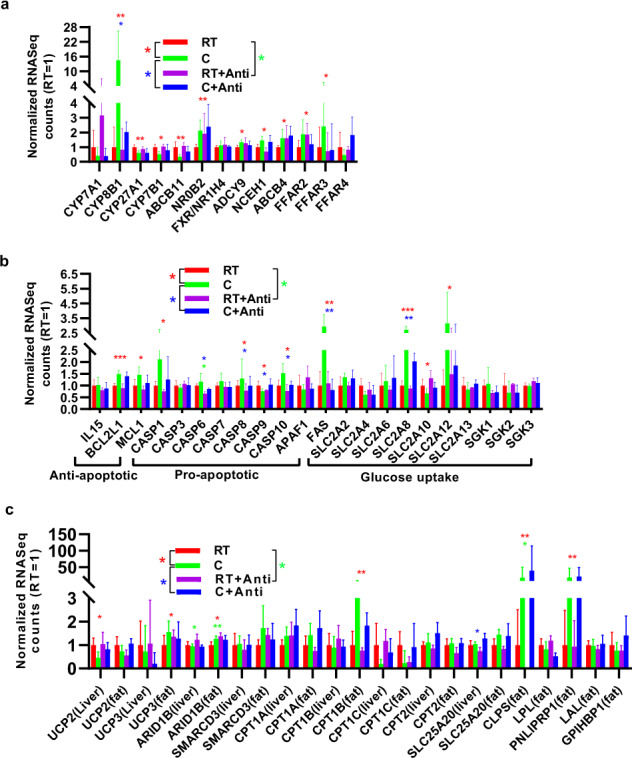
Relative mRNA expression in the liver or fat quantified by RNA-seq. **a** Genes involved in bile acid and short-chain fatty acid metabolism in the liver. **b** Genes involved in antiapoptotic, proapoptotic, and glucose uptake effects in the liver. **c** Genes involved in the balance of thermogenesis and fat metabolism. The data are the means ± SEMs (*n* = 3 or 5 per group). Statistical significance was determined using Wilcoxon test (*p < 0.05, **p < 0.01, ***p < 0.001).

To uncover the mechanism underlying the microbiota-liver-fat cross-talk responsible for the energy balance, we performed deep sequencing of the transcriptome from the liver and fat in the four groups. The expression levels of the gene transcripts are shown in Supplementary Data 4. Pathway enrichment analysis revealed that changes in pathways involved in butanoate, propanoate, and pyruvate metabolism, pentose and glucuronate interconversions, and glycolysis/gluconeogenesis related to carbohydrate metabolism were decreased in the liver under cold stress compared with RT (Supplementary Fig. 10a). Glycerolipidsc, fatty acid degradation and metabolism, ascorbate and alternate metabolism, and retinol metabolism related to vitamins were also decreased in the liver under cold stress (Supplementary Fig. 10a). Tryptophan metabolism, valine, leucine, and isoleucine degradation pathways related to amino acid metabolism and steroid hormone biosynthesis pathway related to hormone biosynthesis were also decreased under cold stress compared with RT (Supplementary Fig. 10a). Most signaling pathways, including the cytosolic DNA-sensing, TNF, NOD-like receptor, and IL-17 signaling pathways, were enriched under cold stress in comparison to the levels under RT. Pyrimidine metabolism, RNA degradation, RNA polymerase, proteasome, and ribosome biogenesis in eukaryotes were also enriched under cold stress, compared with RT pigs (Supplementary Fig. 10b). The immune signaling pathways in fat were decreased under cold stress compared with RT (Supplementary Fig. 10c). However, in fat, the pathways related to the energy supply, including the PPAR, cGMP-PKG, AMPK, adipocytokine, and calcium signaling pathways, were enriched under cold stress compared with RT. Regarding substrate metabolism, features of the energy supply, including protein digestion and absorption, vitamin digestion and absorption, glycerolipid metabolism, fat digestion, and absorption, were enriched under cold stress, compared with RT (Supplementary Fig. 10d). We found that the levels of CYP8B1, which is involved in the main bile acid synthesis pathway, in the liver was 16 times higher under cold stress than under RT (Fig. 6a). However, the increasing trend was suppressed, and significant differences were found between the cold and cold+antibiotics groups, indicating that the gut microbiota might play a very important role in manipulating the bile acid metabolism pathway (*P* < 0.05). The NR0B2 and FXR receptors, which can combine with bile acid to regulate the bile acid cycle, were enriched in the liver under cold stress compared with RT. The genes ADCY9, NCEH1, and ABCE4, which are involved in the bile secretion pathway, were also enriched under cold stress. ADCY9 encodes neutral cholesterol ester hydrolase 1, which is responsible for transferring cholesterol ester to cholesterol and is an important precursor for bile acid synthesis. However, the genes responsible for secondary bile acid biosynthesis in the gut microbiome, including conjugated bile acid hydrolase (cbh), bile acid-coenzyme a ligase (baiB), and 3-dehydro-bile acid delta (4,6)-reductase (baiN), were not influenced by cold stress (*P* > 0.05, Supplementary Fig. 11). Other important microbial metabolites include SCFAs, which play a very important role in host energy balance regulation through the receptors FFAR2, FFAR3, and FFAR4. We found that the genes FFAR2 and FFAR3 were upregulated by cold stress in the livers of pigs (*P* < 0.05, (Fig. 6a), while FFAR4 was slightly downregulated. The heatmaps showed the variability across samples (Supplementary Fig. 12)

Expression of the BCL2L1 and MCL1 genes involved in antiapoptotic functions increased in the livers of pigs under cold stressed compared with RT (Fig. 6b). The expression of the proapoptotic genes CASP6, CASP8, CASP10, and FAS also increased under cold stress. However, TNF signaling, which was activated by bacteria, promotes apoptosis (Fig. 6b). Thus, these proapoptotic genes were significantly suppressed in all microbiota-depleted pigs (Fig. 6b). The FAS, SLC2A8B, and SLC2A12 genes involved in glucose transporters were upregulated under cold stress, indicating that glucose metabolism could be enhanced by the microbiota under cold stress.

Additionally, insulin resistance was higher under cold stress than under RT, which was consistent with a previous insulin resistance experiment. Fat is important for thermogenesis, especially for UCP genes. However, the UCP1 gene is enriched in BAT and is absent in pigs. We also investigated whether the level of the UCP gene involved in thermal energy regulation was significantly different under cold stress in the liver and fat (Fig. 6c). The results revealed decreased UCP2 gene levels under cold stress in the liver; in contrast, the UCP3 gene level increased in fat under cold stress compared to that at RT, which suggested that fat might represent an important energy source for piglets under short-term cold stress through UCP3-related pathways. The genes ARID1B, SMARCD3, CPT1A, CPT1B, and SLC25A20, which are involved in fat metabolism for thermogenesis, were enriched in fat under cold stress (Fig. 6c). In particular, CPT1B, which is necessary for the ability to use fat as energy supply through β-oxidation, was enriched under cold stress at levels 4 times those at RT. Expression of the CLPS and PNLIPRT1 genes responsible for fat metabolism increased 18-fold under cold stress; however, no difference was found between cells treated with or without antibiotics under cold stress (Fig. 6c).

### Cold stress changes the turnover of the total, abundant, and rare ASV fractions

**Fig. 7:**
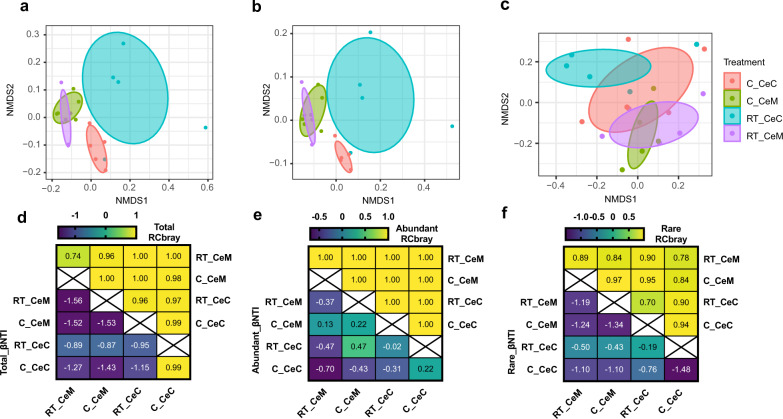
Taxonomic distribution of total, abundant, and rare ASVs in the different groups. ASVs with average relative abundances ≥1% and <0.1% were assigned as abundant ASVs and rare ASVs, respectively. Nonmetric multidimensional scaling (NMDS) plots of beta-diversity were generated from **a** total ASVs. **b** Abundant ASVs. **c** Rare ASVs. Heatmap of beta nearest taxon index (βNTI) and the Raup–Crick metric based on the relative abundances of the ASVs (RC_Bray_) of total, abundant, and rare communities are shown in (**d**–**f)**, respectively. RT_CeM and C_CeM represent the cecal epithelial surface in the RT group and cold-stressed groups, respectively, while RT_CeC and C_CeC represent the cecal content in the RT and cold-stressed groups, respectively.

We calculated the βNTI and RC_Bray_ to determine the assembly processes that drove the microbial community composition under different conditions (Fig. 7). In the total, abundant, and rare ASV fractions, the βNTI values were all between −2 and 2, indicating that the stochastic process was critical. With further analysis, the RC_Bray_ values of the microbial communities in the most abundant and total ASVs, except RT_CeM, were >0.95, suggesting that dispersal limitation predominantly governed the microbial communities. Conversely, the RC_Bray_ values of the microbial communities of the rare ASVs were only found to be >0.95 in C_CeM and C_CeM-RT_CeC, and in the other pairwise samples, the RC_Bray_ values were <0.95, reflecting that drifting alone (weak selection) determined the composition of the microbial community.

## Discussion

**Fig. 8:**
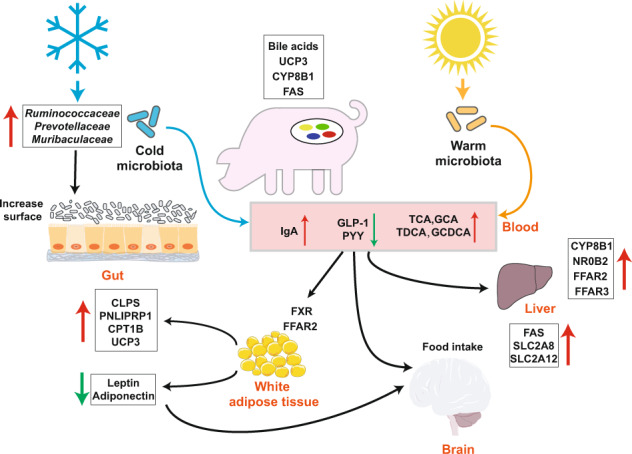
Paradigm summarizing the crosstalk between the gut microbiota and the host via the gut microbiota-blood-liver and fat axes in mediating host thermogenesis under cold stress. FFAR2, free fatty acid receptor 2; FFAR2, free fatty acid receptor 3; UCP3, uncoupling protein 3; GLP-1, glucagon-like peptide-1; PYY, peptide YY; SCFAs, short-chain fatty acids; TCA, taurocholic acid; GCA, sodium glycocholate hydrate; TLCA, taurolithocholic acid; TDCA, taurodeoxycholic acid; CDCA, chenodeoxycholic acid; GCDCA, glycochenodeoxycholic acid; CYP8B1, cytochrome P450, family 8, subfamily b, polypeptide 1; NR0B2, nuclear receptor subfamily 0, group B, member 2; FAS, fatty acid synthase; SLC2A8, solute carrier family 2 (facilitated glucose transporter) member 8; SLC2A12, solute carrier family 2 (facilitated glucose transporter) member 12; FXR, farnesoid X receptor; CLPS, colipase; CPT1B, carnitine palmitoyltransferase 1B. The elements, such as snowflake, sun, liver, and brain, were used from BioRender-biorender.com platform.

The gut microbiota is shaped by diet, environmental conditions, and physiological, host, and social influences, which have dual effects on the host and gut microbiota^21^. In the present study, we investigated the bidirectional effects between the gut microbiota and the host piglets in regulating thermogenesis (Fig. 8). The composition, structure, and functions of the microbiota of the cecum and its mucosal surface were affected by cold stress, and there were correlations between the gut microbiota and thermogenesis through metabolites regulating the signal and gene expression in the liver and fat. After the host experienced cold stress, glucose was first mobilized as energy. However, when cold stress was prolonged, the pool of host glucose was consumed; hormones related to glucose metabolism, such as cortisol, T3, and T4, were depleted, ﻿and fat metabolism in the host was increased to supply energy.

Cold stress increases some secondary bile acids through metabolites in the blood and their receptors (NR0B2 and FXR), activating the cGMP-PKG, PPAR, AMPK, and calcium signaling pathways in fat and enriching genes involved in fat degradation, including CLPS, PNLIPEP1, CPT1A/B, and UCP3, thus leading to thermogenesis in the host. These data suggest that gut microbiota-bile acid interactions occur through the host’s comprehensive signaling network via fat-mediated host energetics and thermogenesis during cold stress in piglets.

Cold stress could significantly suppress the production of GLP-1 and PYY, which regulate food intake and the levels of which normally peak at 20 min after food intake^22^, indicating that the negative feedback regulating food intake was weakened under cold stress and allowed piglets to consume more food to produce heat and maintain a normal body temperature. Cold stress increased insulin resistance; however, after depletion of the microbiome, insulin resistance was lowered, indicating that the gut microbiota played an important role in this function, which is consistent with previous reports in mice under cold stress^10,11,14^. This finding suggested that a broad range of antibiotics that interrupted the piglet intestinal microbiota would influence glucose uptake and metabolism in the host. Interesting results from our study further demonstrated that serum hormones involved in energy metabolism, including T3, T4, cortisol, leptin, and adiponectin, were suppressed in pigs exposed to cold stress. Leptin and adiponectin are secreted by fat^23^. A decrease in the leptin level might weaken the suppression of food intake through the pro-opiomelanocortin (POMC) pathway and decrease energy exhaustion, indicating that pigs could store more energy to resist cold stress^22,24^. However, T3, T4, cortisol, and adiponectin, which play important roles in energy exhaustion, were found to be enriched under cold stress in most previous studies^11,12^. The reason for this phenomenon might be that the piglets had low energy stores, and under cold stress, the energy was not adequate and was exhausted, so liver function was suppressed to decrease T3 and T4 secretion through the hormonal or leptin pathways^25^. Cold stress increased the weight of the critical immune organs in pigs, that is, the spleen and thymus, while depletion of the gut microbiota weakened this effect, indicating that the gut microbiota might play a very important role in immune functions. The levels of IgA and IL-18 also increased under cold stress, further confirming that short-term cold stress enhanced the host immune response and that the gut microbiota might play a key role in immune function^26^. Cold stress increased duodenal mucosal thickness, villus length, crypt depth, and cecal mucosal thickness, which might because the piglets had an increased intestinal surface, to which strengthened glucose uptake for energy supply. Cold stress could increase the duodenal villus length and mucosal thickness in pigs, but the use of antibiotics further weakened these effects. These results suggested that the intestinal morphological response to the environment was also determined by intestinal microbiota homeostasis. A previous report showed that mice had an increased intestinal surface after cold microbiota transplantation, which strengthened glucose uptake for the energy supply^11^.

In pigs under cold stress, the diversity of the gut microbiota increased, and the Shannon index was significantly higher than that under RT (Fig. 5c). However, most previous studies in mice have shown that diversity is not influenced by cold stress^11,12,15,27^, and cold stress also decreases alpha diversity, including the Shannon index and observed ASVs. However, after 4 weeks of cold stress in male Brandt’s voles, the alpha diversity of the cecal microbiota increased^12^, indicating that changes in the diversity of the gut microbiota under cold stress might be affected by the breed of animal and the duration of exposure to cold stress. The most abundant phyla in the cecum or its mucosal surface in pigs were Firmicutes, Bacteroidetes, Proteobacteria, and Actinomycetes, accounting for up to 95% of the microbiota, which is consistent with findings in other mammals, such as mice^11^ and humans^28^. At the phylum level, however, not all bacteria were affected by cold stress, and at the mucosal surface, only Firmicutes showed an increasing trend (*P* = 0.071), consistent with a previous study conducted in mice under cold stress; additional differences have been found at the family, genus, and species levels^27^. However, the number of different ASVs from the mucosal surface of the cecum was higher under cold stress than under RT, suggesting that the microbiota in the cecum might be strongly affected under cold stress or that the microbiota on the mucosal surface was limited. Many studies have shown that environmental temperature affects the gut microbiota of animals^12,27^. *Prevotellaceae* and *Ruminococcaceae* were the most abundant ASVs under cold stress compared with RT, consistent with a previous study in ﻿male Brandt’s voles and mice under cold stress^10^. These bacteria may play very important roles in the fermentation of residues, including complex carbohydrates that cannot be digested in their original form in the intestine of the hindgut^29^. We also found a higher abundance of *Muribaculaceae* in both the cecum and on the mucosal surface under cold stress than at RT. *Muribaculaceae* is the new name of family S24-7 (phylum Bacteroidetes) and includes versatile microorganisms that are involved in complex carbohydrate degradation and have been defined as the main mucosal sugar utilizers that reduce *Clostridiodes difficile* colonization^30,31^. ﻿*Rikenellaceae* and *﻿Lachnospiraceae*, which were enriched after cold acclimation in pigs, could potentially also have the ability to utilize mucosal sugars and may be associated with host health^31–34^. *Lachnospiraceae* is also an important butyrate producer that impacts colonization resistance through antibiotic expression or intestinal acidification and influences host mucosal immune cells and enterocytes^34^. ﻿*Alloprevotella*, which was enriched on the mucosal surface of pigs after cold acclimation, is similar to *Prevottella* and correlated with a low cardiovascular disease risk^35^. ﻿*Erysipelotrichaceae* was enriched at the mucosal-surface of pigs after cold exposure. However, *Erysipelotrichaceae* was depleted in mice after cold exposure, and this difference could be attributed to the distinct positions of the gut microbiota samples^35^. Some bacteria in *Erysipelotrichaceae* have been reported to be colitogenic strains^36^ and to be correlated with host health^37^. βNTI analysis provides a top-down view of the assembly of the microbiome at the ecological level. These results reflect the change in assembly types of all, dominant and rare, microbiomes under cold stress. Furthermore, we can extract or assume the response of microbiomes with different relative abundances and the main factors influencing these changes^38,39^.

Microbe–metabolite vectors correlation analysis was used in our study to determine the correlation between microbiomes and serum metabolites^20^. ﻿Taurolithocholic acid (TLCA), ﻿taurodeoxycholic acid (TDCA), and taurocholic acid (TCA) were correlated with ﻿the *Rikenellaceae* RC9 gut group and ﻿*Muribaculaceae*. ﻿The glycocholic acid (GCA) and glycochenodeoxycholate (GCDCA) levels were elevated in serum under cold stress compared with the levels at RT (Supplementary Fig. 8). GCA has the highest FXR receptor affinity^40^. However, we did not find a strong correlation between bacteria and these bile acids, possibly because the main bacterial genera of the gut microbiota involved in bile acid metabolism occurred in the small intestine^20^. However, the families *Ruminococcaceae* and *Prevotellaceae* were the main bacteria that were correlated with metabolites in serum. ﻿Cold exposure of the microbiota could induce an increase in the expression of the bile acid synthesis gene CYP8B1 in the liver, which is consistent with previous studies^10^. However, we did not find a significant difference after cold exposure in the level of the important gene CYP7A1, which has been reported to increase after cold exposure in mice^10,14^. The receptors and genes involved in bile acid metabolism including NR0B2, FXR, ADCY9, NCEH1, and ABCB4, were enriched after cold exposure, which is consistent with previous studies^10,14^. The strength of bile acid metabolism may contribute to host energy homeostasis^14^. SCFAs can also act as signal metabolites via the G-protein-coupled receptors FFAR2, FFAR3, HCA2, HCA1, and SUCNR1 during appetite regulation and thermoregulation^23,41^. Expression of the FFAR2 and FFAR3 genes was elevated in the liver under cold stress compared with RT, which is consistent with a previous study, and FFAR2 expression in fat was increased in ﻿male Brandt’s voles (*Lasiopodomys brandtii*)^12^. However, a previous study showed that the levels of FFAR2 and FFAR3 did not differ the following exposure to different antibiotic groups to deplete the gut microbiota^27^. The same result was obtained in the present study for FFAR3, but the expression of FFAR2 was increased at RT and decreased under cold stress after antibiotic treatment. These differences might require further analysis for validation. Increasing evidence indicates that SCFAs can regulate appetite by increasing levels of glucagon-like peptide-1 (GLP-1)/fasting PYY, a peptide hormone that reduces appetite^23^. However, we found that the levels of GLP-1 and PYY decreased under cold stress, which might increase appetite and thus food intake to maintain energy requirements. Fat is important for energy supply when carbohydrates or glucose are insufficient, especially in a cold environment^7^. Expression of the fat lipolysis genes CLPS and PNLIPRP1 was elevated under cold stress compared with RT. After fat lipolysis, fat must be transferred into mitochondria for energy supply through hepatic fatty acid oxidation by CPT1A/B, the level of which increased in the fat of pigs under cold stress in our study, consistent with a previous report on CPT1A in mouse livers under cold stress^10^. The UCP gene was the last and key gene for heat supply. Most studies have shown that in most mammals exposed to cold stress, fat can be transferred to BAT by UCP1, and the gut microbiota also plays a very important role in this process^10,12,27^. However, pigs lack UCP1 genes and cannot brown WAT to achieve a balance in thermogenesis^42–44^. However, using RNA-seq, we found that the levels of UCP2/3 and UCP3 were elevated under cold stress compared with the levels at RT, indicating that UCP3 might play a very important role in supplying heat by oxidizing fat in pigs. This result is consistent with the finding that UCP3 expression is elevated under cold stress in Tibetan pigs^45^, indicating that UCP3 also plays a very important role in thermogenesis in pigs.

In conclusion, cold stress is a normal environmental stress in piglets and disturbs the balance of their gut microbiota, affecting host metabolic physiology. The present study illustrated the relationship and possible interacting pathways between the host piglets and microbiota under cold stress. Our data demonstrate that cold stress induced a wide range of physiological changes, such as increased insulin resistance and IgA levels and decreased cortisol, T3, T4, leptin, adiponectin, GLP-1, and PYY levels. Cold stress also caused significant alterations in the content and microbiota of the cecum and its epithelial surface, which was accompanied by an increase in the levels of certain secondary bile acids in serum in piglets exposed to cold stress. The most significantly correlated metabolites in serum were associated with the ASVs belonging to *Ruminococcaceae*, *Prevotellaceae*, and *Muribaculaceae*. Moreover, transcriptomic results showed higher expression of CYP8B1, FXR, FFAR2, and FFAR3, which are related to bile acid and SCFA metabolism, in the liver under cold stress compared with RT. In addition, the levels of the fat lipolysis genes CLPS, PNLIPRP1, CPT1B, and UCP3 were significantly increased in the fat of piglets exposed to cold stress. However, antibiotic-treated piglets showed a weakened or strengthened cold tolerance phenotype, indicating a link between the host response to cold stress and regulation by the gut microbiota. These findings suggest that gut microbiota-blood-liver/fat axes mediate host thermogenesis under cold stress via metabolites produced by bacteria and the host. These results provide considerable new insight into the communication between piglets and the gut microbiota to improve the survival or health status of piglets experiencing cold stress.

## Methods

### Study sites and sample set

The breeding experiment was carried out at the experimental base in Zengcheng District, Guangzhou city, Guangdong Province. After weaning for one week, a total of 20 piglets were randomly divided into the room temperature (RT), room temperature with antibiotics (RT + antibiotics), cold stress (C) and cold stress with antibiotics (cold+ antibiotics) groups with five replicates per group according to body weight. Piglets were reared in turning carts, with each turning cart divided into pens. Each turning cart included 3 experimental animals. Piglets were provided with feed and water ad libitum; the remaining feed in the trough was cleaned up with new feed added at 08:00 and 18:00, and the cart and environment were simultaneously cleaned. After one week of adaptation, the RT + antibiotics and cold+antibiotics groups received drinking water containing neomycin (227.30 mg/day), streptomycin (113.60 mg/day), vancomycin (113.60 mg/day), metronidazole (227.30 mg/day), bacitracin (2.27 g/day), ciprofloxacin (284.10 mg/day), ceftazidime (227.30 mg/day), gentamicin (396.40 mg/day), and penicillin (227.30 mg/day) until the end of the experiment. During the depletion period, 2 piglets in the RT + antibiotics group were removed because of health problems. Piglets in the nonantibiotic group did not receive any drugs or antibiotics, and the health status of all piglets was recorded. One week after receiving antibiotics, the cold stress groups were exposed to a low temperature of 18 °C for 48 h. The experimental design and procedures were conducted according to the institutional guidelines for the care and use of experimental procedures involving animals were approved by the Animal Experimental Committee of South China Agricultural University (SYXK2014-0136).

Finally, we collected samples from 18 piglets, including 5 piglets in the RT group, 5 piglets in the cold stress group, 5 piglets in the cold stress group treated with antibiotics, and 3 piglets in the RT group treated with antibiotics. Blood was collected from the anterior vena cava of each piglet to extract serum for nontargeted metabolomics analysis, determine the composition of bile acid, and measure other parameters. Each intestinal segment was punctured and carefully cut. Cecal contents and epithelial surface samples were collected for 16S rRNA and metagenomic analysis and SCFA measurement. Then, the remaining contents were removed, and the cecal wall was cut and washed with 4 °C normal saline. A sterile glass slide was used to gently scrape the intestinal mucosa, and other tissue samples (liver, spleen, and thymus) were first weighed and collected in the same way. All utensils used in the sample collection process were sterilized, and each group of samples was divided into three sterile centrifuge tubes, quickly placed in liquid nitrogen, transferred to the laboratory, and stored at −80 °C for testing. All samples from each pig were collected within 30 min after slaughter. The details of the samples used for the subsequent analyses are listed in Supplementary Fig. 1.

### Measurement of indicators of in serum

The blood samples were centrifuged (5000 × *g*) at RT after its natural coagulation for 10–20 min. Serum IgA, IgG, IL-6, IL-10, IL-18, and NF-kB were quantified via a radioimmunoassay using a Porcine ELISA Kit (Shanghai MLBIO Biotechnology Co. Ltd, Shanghai, China) according to the manufacturer’s instructions. Serum glucose, insulin, cortisol, leptin, adiponectin, T3, T4, GLP-1, and peptide YY (PYY) were quantified via radioimmunoassay using an ELISA Kit (Nanjing Jiancheng Bioengineering Institute, Nanjing, China) according to the manufacturer’s instructions. The plots were generated using ggplot2, and the significance level of the difference between unpaired groups was determined with the Wilcoxon test (*p < = 0.05; **p < = 0.01; ***p < = 0.001) using ggpubr in R (4.1.1).

### Histology

Freshly harvested duodenal and cecal tissue were fixed in 10% buffered formalin for 24–48 h at RT, washed twice with PBS, and stored in PBS at 4 °C before paraffin embedding. The tissue was cut and stained with H&E using standard techniques. Each slice from each group was photographed at 40 times the visual field, and five intact villi were selected to measure the mucosal thickness, villus length, and crypt depth, which were analyzed using Image-Pro Plus 6.0 processing software (Media Cybernetics, Inc., Rockville, MD, USA).

### 16S rRNA gene amplicon sequencing

Approximately 200 mg of cecal contents and epithelial surface samples from the nonantibiotic groups were used for total DNA extraction using the QIAamp Power Fecal Pro DNA Kit (QIAGEN GmbH, QIAGEN Strasse 1, 40724 Hilden, Germany) following the manufacturer’s instructions. The DNA samples were stored at −20 °C for 16S rRNA gene amplicon and metagenomic sequencing. The quantified library was sequenced with the Illumina HiSeq 2500 platform, and the V4 hypervariable region was PCR-amplified with the primers 515F (5′-GTGCCAGCMGCCGCGGTAA-3′) and 806R (5′-GGACTACHVGGGTWTCTAAT-3′). PCR amplifications were performed using 25 µL reaction mixtures containing 11 µL of PCR-grade water, 10 µL of 5′ PRIME HotMasterMix, 3 µL of DNA template, and 0.5 µL of each primer (10 µM). PCR amplifications were conducted using the following program: predenaturation at 94 °C for 5 min; 30 cycles of amplification, including denaturation at 94 °C for 30 s, annealing at 50 °C for 30 s, and elongation at 72 °C for 30 s; and a final elongation at 72 °C for 10 min. To ensure the efficiency and accuracy of amplification, quantitative and qualitative inspection of the DNA was performed using a Qubit nucleic acid quantitative analyzer and 2% agarose gel electrophoresis. PCR amplification, sequencing, and data quality control were performed by Novogene Co., Ltd. (Beijing, China). Raw reads were uploaded into Quantitative Insights in Microbial Ecology (QIIME2.2019.10)^46^ and qualitatively trimmed. Quality monitoring was used to filter out unqualified reads and sequences (<25 mass score and >225 bp length) to obtain clean reads. DADA2^47^ was used to perform dereplication (dereplication, equivalent to 100% similarity clustering) and obtain amplicon sequence variants (ASVs). The parameters of DADA2 were set at -p-trim-left-f 0, -p-trim-left-r 0, -p-trunc-len-f 200, and -p-trunc-len-r 200, and the numbers of reads with nonchimeric sequences ranged from 29,860 to 62,832, with an average of 51,490. In total, 2501 ASVs were identified in all samples, based on 1,061,089 total reads, with a mean number of reads of 53,054 per sample. The rarity curves of the cecal contents and epithelial surface DNA samples were basically stable, and the sampling depth was sufficient to describe the microbial community (Supplementary Fig. 2). The feature table, map file, and classification tree analyzed by QIIME2.2019.10 were imported into the Phyloseq (1.22.3)^48^ platform using qiime2r to calculate the alpha diversity, including the Chao1, Shannon, and observed species indices. Two methods, using unweighted and weighted UniFrac distances, were used to calculate the beta diversity of the bacterial flora in the samples. Using permutational multivariate analysis of variance (PERMANOVA), also known as Adonis analysis, the Bray-Curtis distance matrix was used to decompose the total variance, and the degree to which piglet age explained the gut microbiota structure was analyzed using a linear model. A permutation test was used for significance analysis, with *P* < 0.05 indicating significance. Further analysis was performed for the differences in the composition of the gut microbiota.

For the relative abundance at the bacterial phylum level, the Wilcoxon rank-sum test was used for statistical analysis, and *P* < 0.05 was considered to indicate a significant difference. DeSEq2 (1.32.0)^49^ was used to analyze the differences in bacterial composition at the family and genus levels. The difference analysis parameters were set and the ASVs with a padj (corrected *P*-value) <0.05 showed significant differences at the family and genus levels. In DESeq2, the *p-*value obtained by the Wald test is corrected for multiple testing using the Benjamini and Hochberg method by default.

### Metagenomic sequencing and data analysis

DNA was extracted and subjected to quality testing. The DNA from cecal contents and epithelial surface samples in the nonantibiotic groups was analyzed by metagenomic sequencing. All database construction and sequencing services were provided by Novogene Co., Ltd. (Beijing, China). The kneaddata (0.10.0) tool was used for quality control and to filter the low-quality sequences with default parameters (-trimmomatic-options MINLEN:60 ILLUMINACLIP:/-SE.fa:2:30:10 SLIDINGWINDOW:4:20 MINLEN:50), and bowtie2 alignment against the pig host gene (susScr11) was performed to remove possible contaminating pig DNA^50^. After quality control and filtering of the obtained raw data, for all samples, the clean reads were assembled by Megahit (1.2.9)^51^ in paired-end mode, and then, gene prediction was performed using Prodigal (2.6.3)^52^ with the parameter “-p meta”. A nonredundant gene catalog was constructed using the gene models predicted from all genes by cd-hit-est (4.8.1)^53^ with the parameter “-aS 0.9 -c 0.95 -G 0 -g 0”. Functional assignments of the protein sequences were made based on DIAMOND alignment against eggNOG using eggNOG-mapper (2.0.1)^54^ by taking the best hit with the criterion an *E* value <1e^−5^. To calculate the relative gene abundance, the clean reads from each sample were aligned against the gene catalog using Salmon (1.1.0)^55^, and the normalized counts (TPM values) were used to perform the next difference analysis. The functional description of the orthologous gene corresponding to the searched sequence was the final annotation result. STAMP (2.1.3)^56^ was used to analyze the composition differences between the conditions. After false discovery rate (FDR) correction, *P* < 0.05 was used to the threshold for significance. The aim was to identify strains that play a key role under cold stress. MetaWRAP (1.3.1)^57^, a process of metagenome binning, was used to carry out binning analysis on the determined samples, including data quality control, assembly by Meaghit, binning by Concoct (1.0.0)^58^, MaxBin2 (2.2.6)^59^ and MetaBAT2 (2.12.1)^60^, quantification of the abundance of bins in each sample, and analysis of the composition of bins compared with the GTDB database using GTDB-tk software (1.5.0)^61^. In total, 531 metagenome-assembled genomes (MAGs) were identified across all samples. In addition, 191 were obtained after estimation at a completeness of ≥ 90% and a contamination level of ≤ 5% using CheckM (1.0.12)^62^. The above STAMP method was used to determine the difference between two groups.

### Serum metabolomics

First, serum samples were collected from the nonantibiotic groups, pretreated, and finally analyzed by LC/MS using an Agilent 1290 (Agilent Technologies). The raw data obtained were converted into mzXML format using ProteoWizard (version 3)^63^ software. XCMS (1.42.0)^64^ was then used for retention time correction, peak recognition, peak extraction, peak integration, and peak alignment, with the minfrac set to 0.5 and cutoff set to 0.3. Simultaneously, the substances corresponding to the peaks were identified using an in-house R package and a self-assembled secondary mass spectrometry database. The differences in the identified metabolite data tables in the positive and negative ion states were analyzed using the MetaboAnalyst (4.0)^65^ comprehensive metabolomics analysis platform. The autoscaling method was used to standardize the data, and a statistical analysis module was used to analyze differences in the metabolomics results, including OPLS-DA multivariate analysis, heatmap analysis, and random forest machine learning identification of differential metabolites. A heatmap was used to map the top 30 differential metabolites from the analysis, and the metabolites with significant representative characteristics that played an important role under different conditions were identified by using a machine learning method. The SCFAs in the cecal content and bile acid in serum were also analyzed using GC/MS and LC/MS, respectively. The microbe-metabolite vector (mmvec) was used to confirm the correlation between the microbiota and metabolites^20^.

### Liver and fat transcriptomics

Total RNA from liver and fat samples from all four groups (total of 18 samples per sample type) was prepared using the RNAprep Pure Tissue Kit (Tiangen) according to the manufacturer’s instructions. Then, the total RNA was subjected to poly-A + selection, fragmented by metal-ion hydrolysis, and converted to cDNA using the PrimeScript™ RT reagent Kit with gDNA Eraser (Takara). The cDNA was end-repaired adenylated and ligated with Illumina sequencing adapters. Finally, the fragments were amplified by PCR. The PCR program was as follows: initial denaturation for 10 s at 95 °C, followed by 40 cycles of 5 s at 95 °C and 30 s at 60 °C, one cycle of 10 s at 95 °C and 30 s at 60 °C, and one cycle of 15 s at 95 °C. The quantified library was sequenced with the Illumina HiSeq 4000 platform using 2 × 150 bp paired-end sequencing. We used Fastp (version 0.19.7) to perform a basic statistical analysis of the quality of the raw reads. Paired reads were discarded if either read contained adapter contamination, if more than 10% of the bases in either read were uncertain, or if the proportion of low-quality (Phred quality <5) bases in either read was more than 50%. We used hisat2 (version 2.2.1-3n) to map the pig reference sequence with the default parameters (-base-change A, G; -repeat). For the RNA-seq analysis, we used the Deseq2 method to distinguish the difference between two groups at a *q*-value (adjusted *p-*value) of 0.05. Gene-level read counts were generated using feature counts^66^, and the continuous reference pig assembly (*Sscrofa11.1*) database was used as the reference database^67^.

### βNTI analysis

To evaluate the community assembly processes, the mean nearest taxon distance (MNTD) taxonomic β-diversity metrics (βNTI and Bray–Curtis-based Raup–Crick, RCBray) were calculated. |βNTI| > 2 indicated that the deterministic process was the main factor influencing the microbial community across all samples. To elaborate, a βNTI value < −2 suggested homogeneous selection, and a value >2 indicated variable selection. If |βNTI| was <2, the RCBray was calculated: (1) RCBray > 0.95 means dispersal limitations^68^, (2) RCBray < −0.95 indicated homogeneous dispersal, and (3) |RCBray| <0.95 indicated “nondominant” fractions^38,39^.

### Reporting summary

Further information on research design is available in the Nature Research Reporting Summary linked to this article.

## Supplementary information

Supplementary Information

Supplementary Data

Reporting Summary

## Data Availability

Raw sequence data are deposited in the ENA Sequence Read Archive under accession number PRJEB44118.

## Code availability

Code and detailed information are available on github (https://github.com/mijiandui/Porcine-gut-microbiota-in-mediating-host-metabolic-adaptation-to-cold-stress).
