# Supplementary material for: Porcine gut microbiota in mediating host metabolic adaptation to cold stress
Source: NPJ Biofilms Microbiomes. 2022 Apr 5;8:18. doi: 10.1038/s41522-022-00283-2 (PMC8983680; doi:10.1038/s41522-022-00283-2)
Supplement: Supplementary file 1 — Supplementary Information [file 41522_2022_283_MOESM1_ESM.pdf]

Supplementary Information

For

Porcine gut microbiota in mediating host metabolic adaptation to cold stress

Yu Zhang[1,2,3+], Lan Sun[1,2,3+], Run Zhu[1,2,3], Shiyu Zhang[1,2,3], Shuo Liu[1,2,3], Yan Wang[1,2,3], Yinbao Wu[1,2], Sicheng Xing[1,2,3], Xindi Liao[1,2,3*], Jiandui Mi[1,2,3*]

[1] Guangdong Laboratory for Lingnan Modern Agriculture, South China Agricultural University, Guangzhou 510642, China

[2] National Engineering Research Center for Breeding Swine Industry, College of Animal Science, South China Agricultural University, Guangzhou 510642, China

[3] Guangdong Provincial Key Lab of Agro-Animal Genomics and Molecular Breeding and Key Lab of Chicken Genetics, Breeding and Reproduction, Ministry of Agriculture, Guangzhou 510642, China

[+]These authors contributed equally to this work.

[*] Corresponding author: Dr. Xindi Liao and Dr. Jiandui Mi.

Tel: 86-20-85280279, Fax: 86-20-85280740, E-mail address: xdliao@scau.edu.cn; mijiandui@163.com, Full postal address: College of Animal Science, South China Agricultural University, Wushan Road, Tianhe District, Guangzhou, 510642, P. R. China

Contents

12 Supplementary Figures

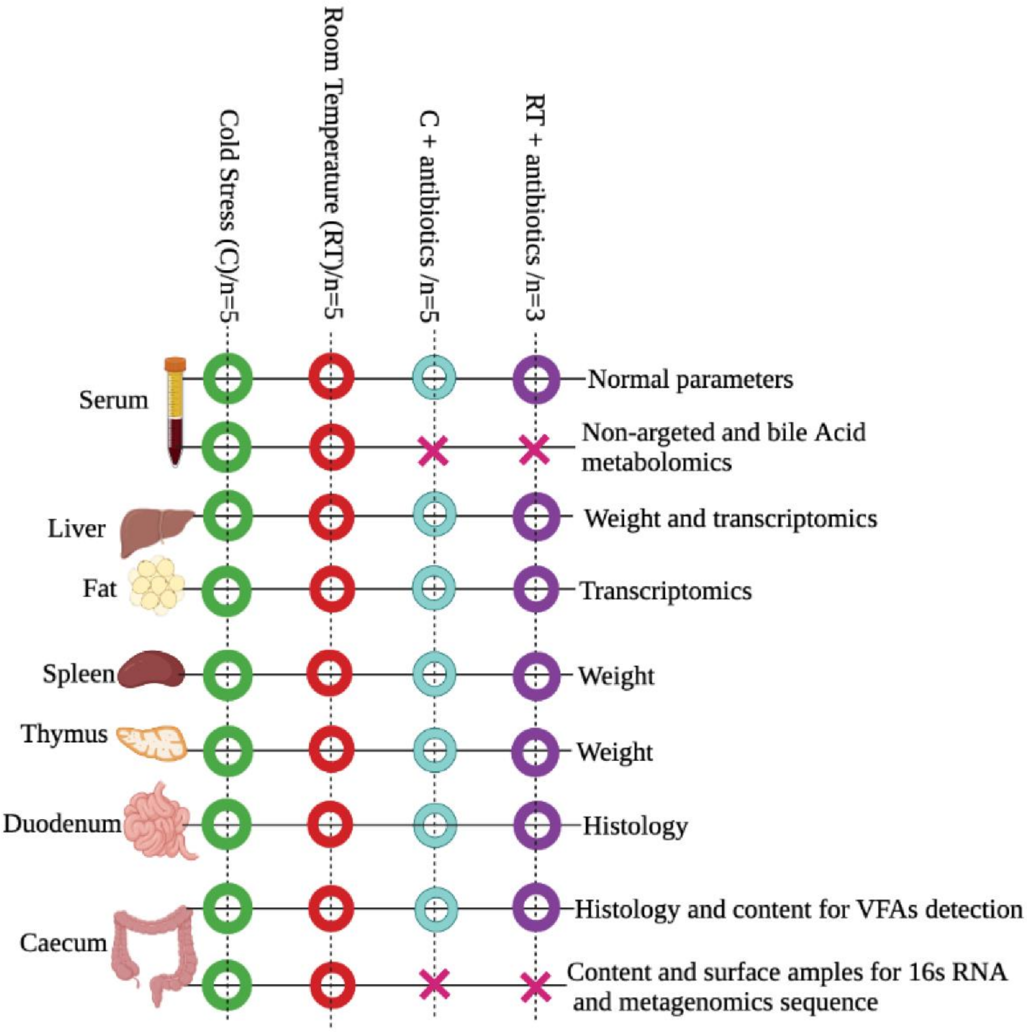

Supplementary Figure 1 Details of sampling and analysis. Circles represent sampling types and analysis items; forks indicate that the corresponding items were not analyzed.

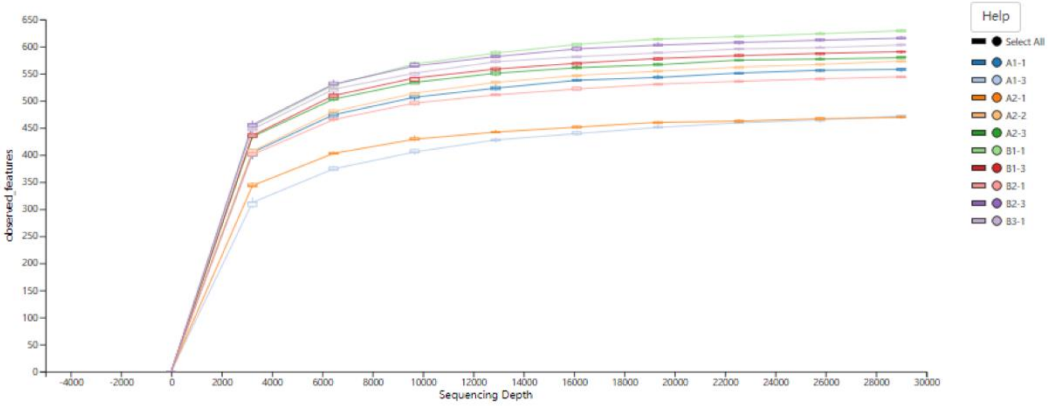

Supplementary Figure 2 Rarity curves for 86 samples.

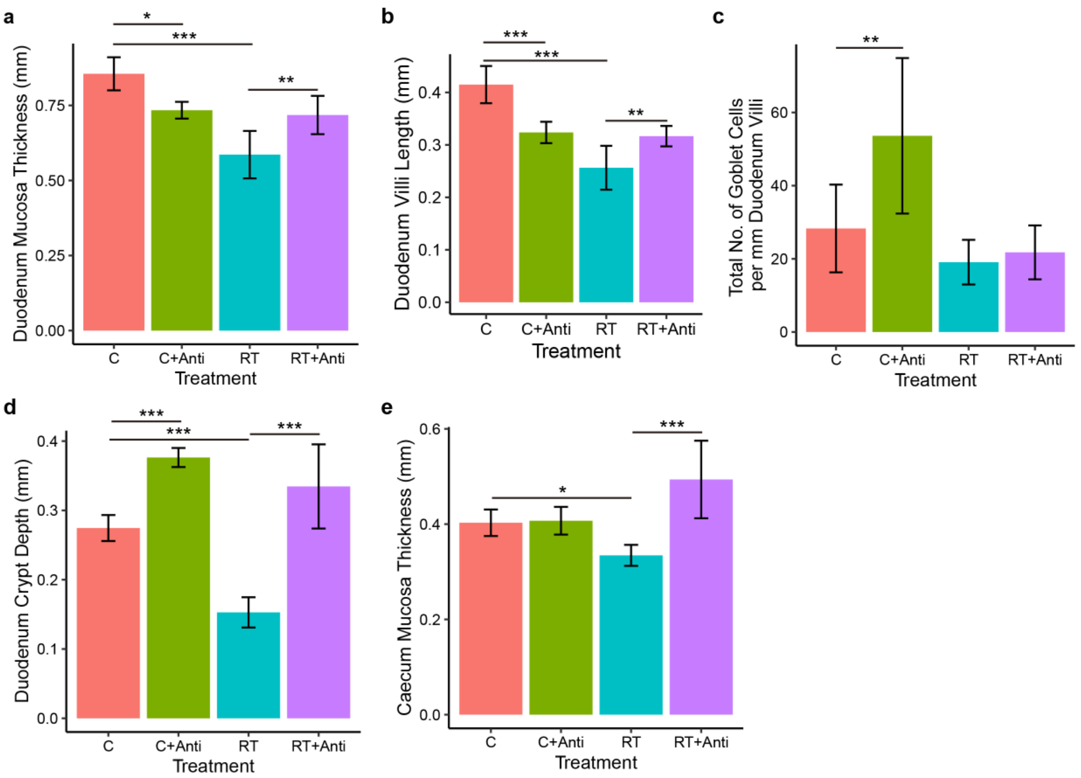

Supplementary Figure 3 Cold-exposed piglets show an increased index for the duodenum. **a** Duodenal mucosa thicknesses. **b** Duodenal villus length. **c** Total number of goblet cells per mm duodenal villi. **d** Duodenal crypt depth. **e** Cecal mucosa thickness. The data are the means ± SEMs. Statistical significance was determined using Wilcoxon test (**p* < 0.05, ** *p* < 0.01, ****p* < 0.001).

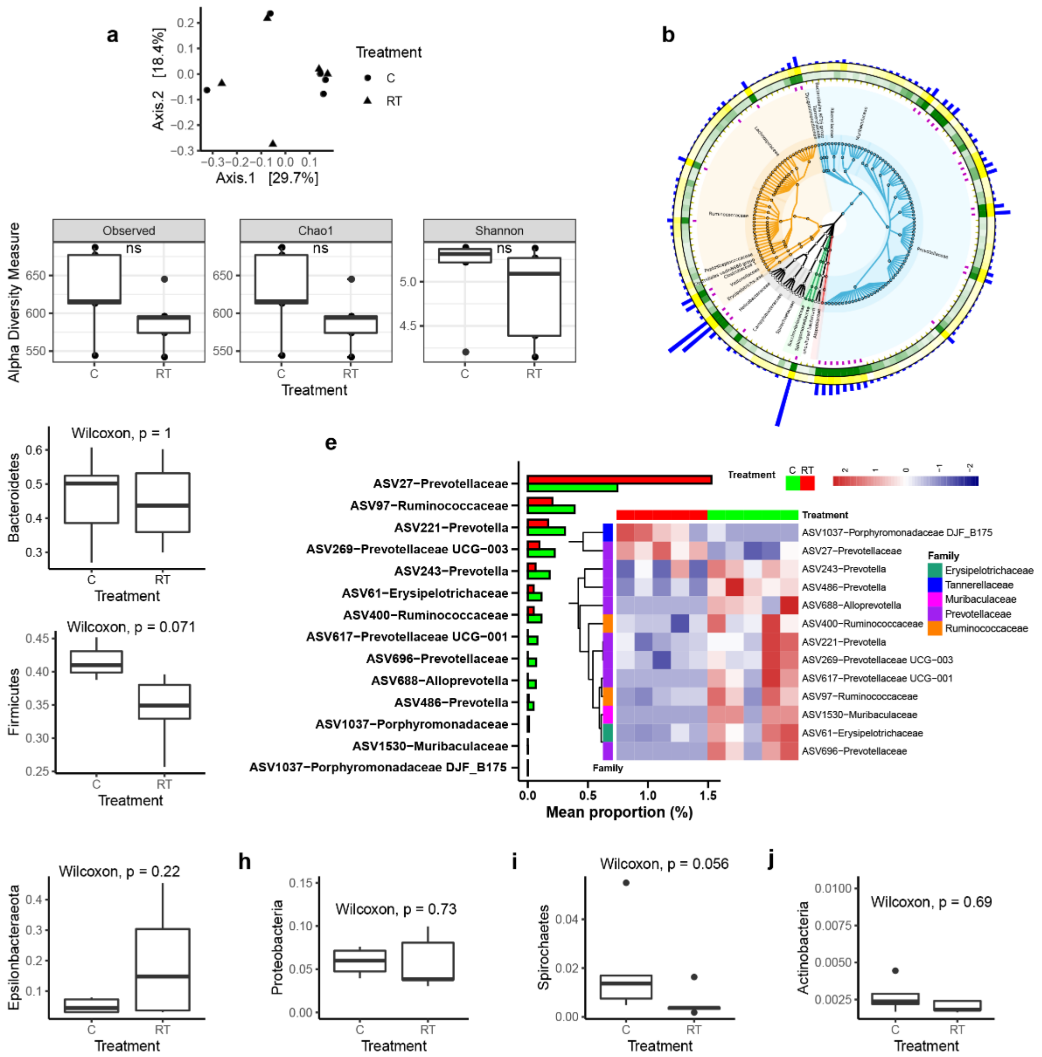

Supplementary Figure 4 Cold exposure changes the composition of the epithelial surface of the cecum. **a** Principal coordinates analysis (PCoA) based on weighted UniFrac analysis. Each symbol represents a single sample of cecal content after 48 h of cold stress (n = 5) or RT (n = 5 per group). **b** Heatmap tree comparing the most abundant ASVs from the cecal content of 48 h cold-stressed animals (n = 5, inner green rings) and RT controls (n = 5, outer yellow rings) and their phylogenetic relationships. The bar represents abundant ASVs. **c** Alpha diversity of ASVs. **d** Boxplot of the microbiota of the cecal content at the phylum level. **e** The different ASVs with bars and heatmaps with $P < 0.05$. **f-j** Boxplot of the microbiota of the cecal content at the phylum level.

The data are the means ± SEMs. Statistical significance was determined using Wilcoxon test (*$p < 0.05$, ** $p < 0.01$, ***$p < 0.001$).

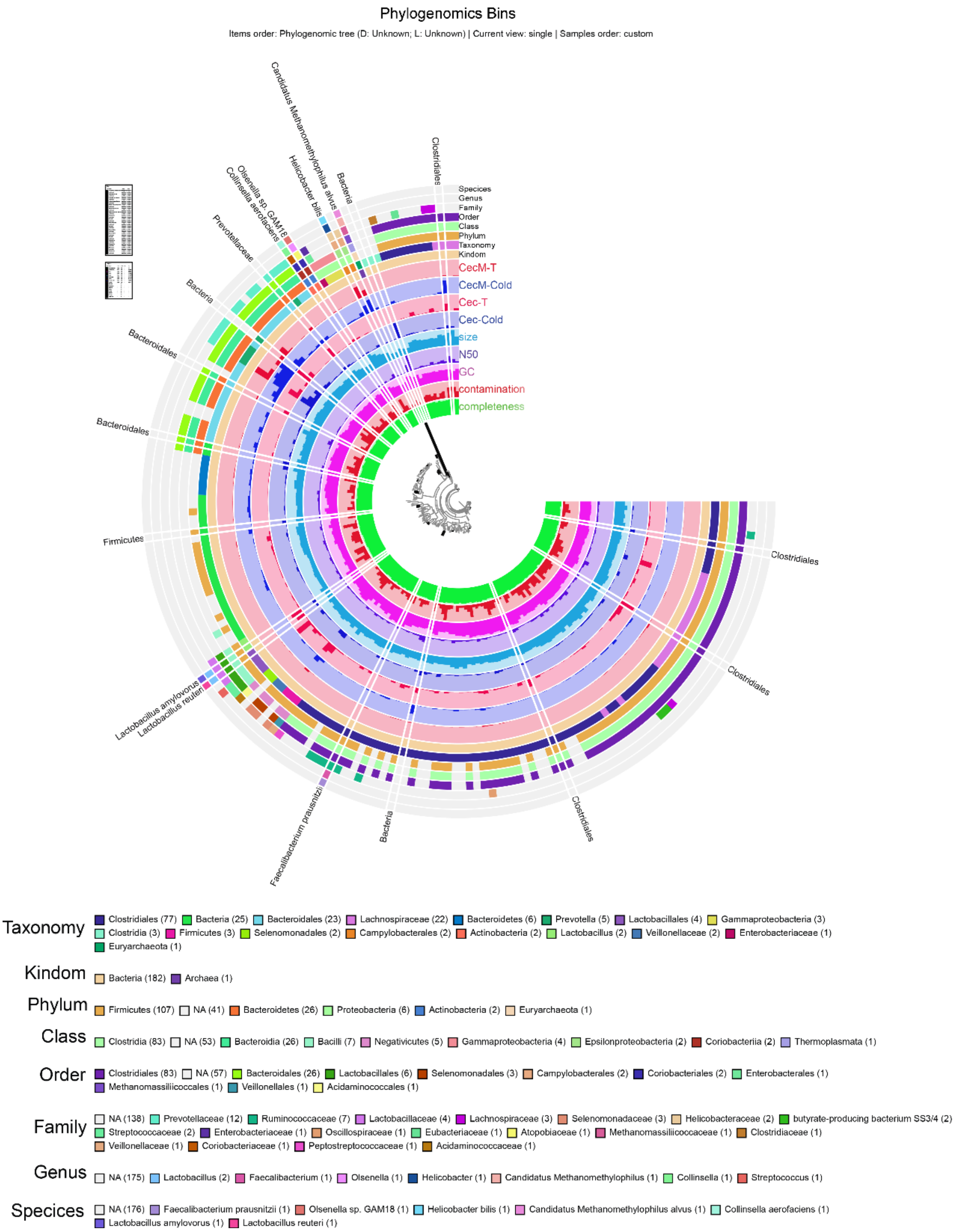

Supplementary Figure 5 Phylogenomic tree with 191 bins with a completeness ≥ 90% and contamination ≤5%.

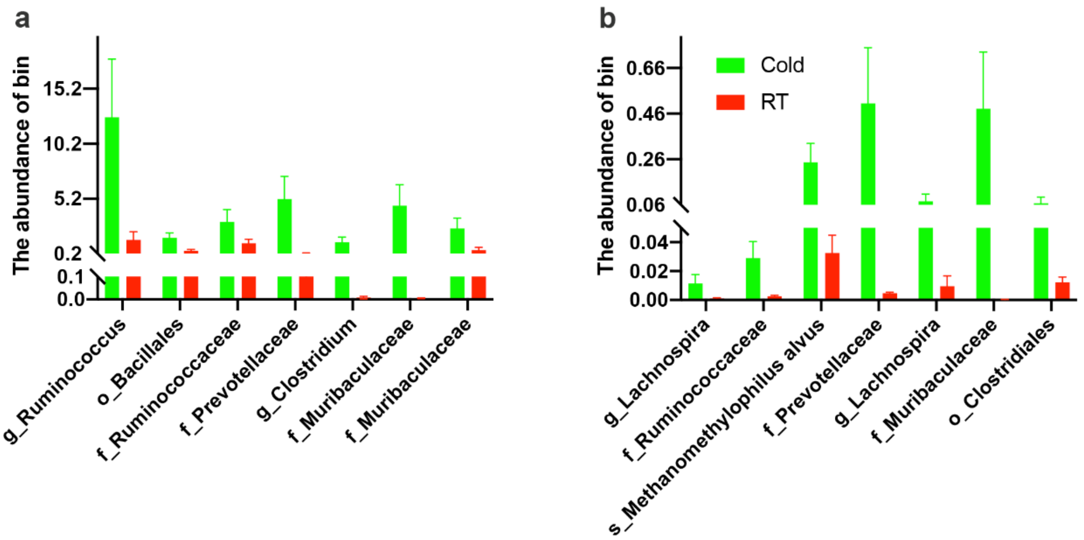

Supplementary Figure 6 Significant bins after STAMP analysis between the RT and cold-stressed groups. The different bins **(a)** in the content and (**b**) on the epithelial surface of the cecum.

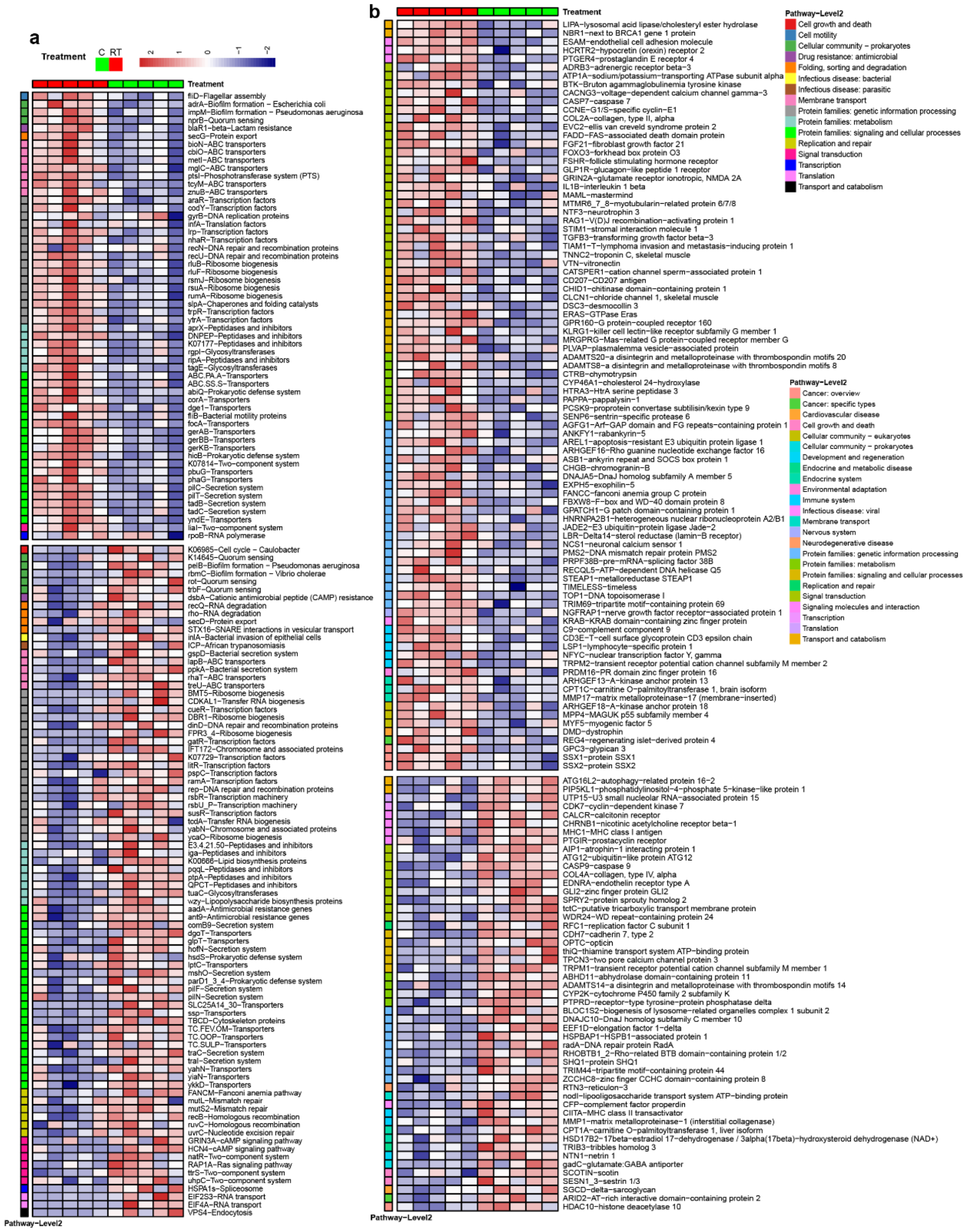

Supplementary Figure 7 Cold exposure changes other functions of the microbiota in the cecum. Heatmap of the differences in the microbiota in the cecum at other functional levels **(a)** in the content and (**b)** on the epithelium surface of the cecum.

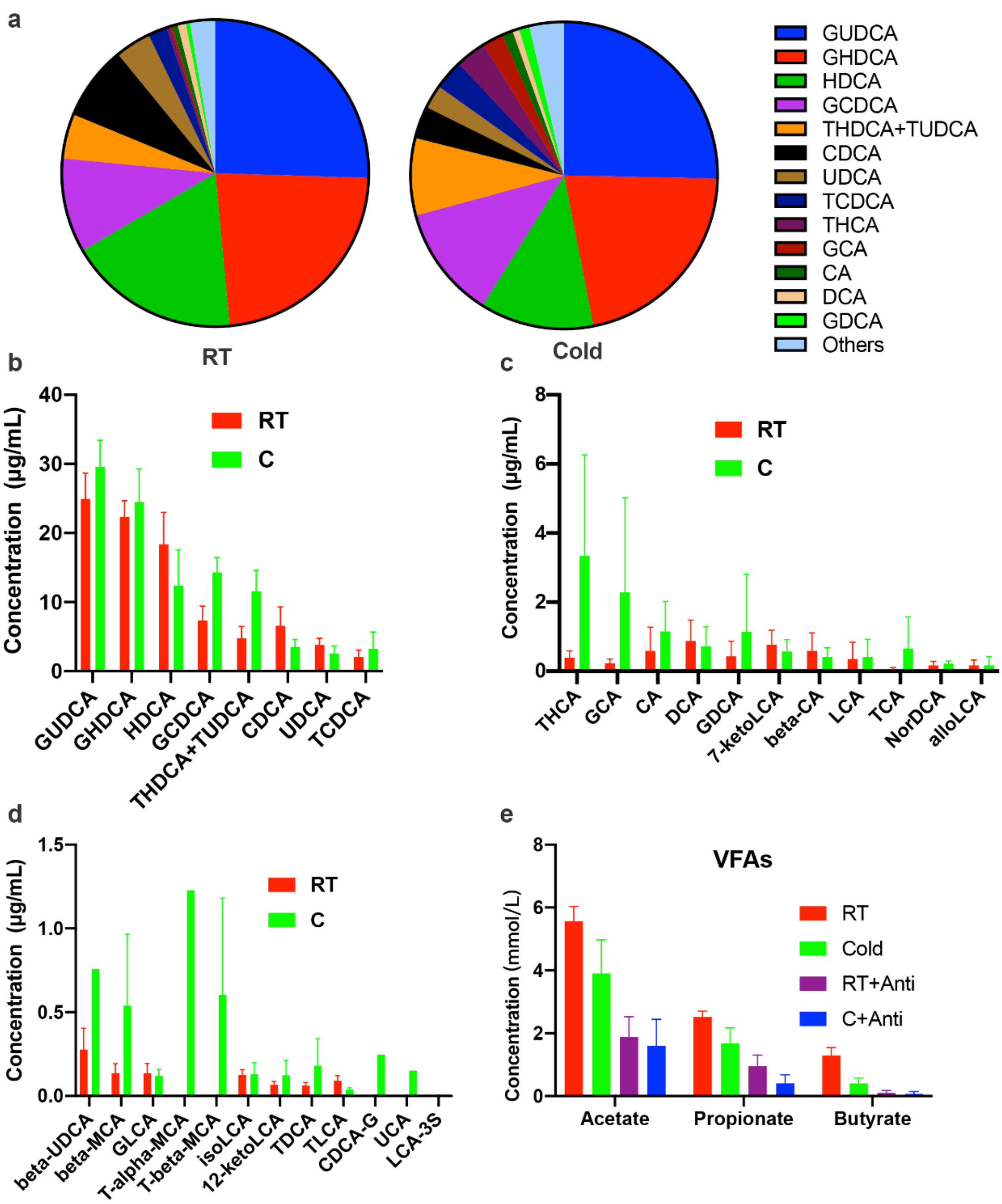

Supplementary Figure 8 Cold exposure changes bile acid concentrations in serum (**a-d**) and SCFA levels (**e**) in the cecal content.

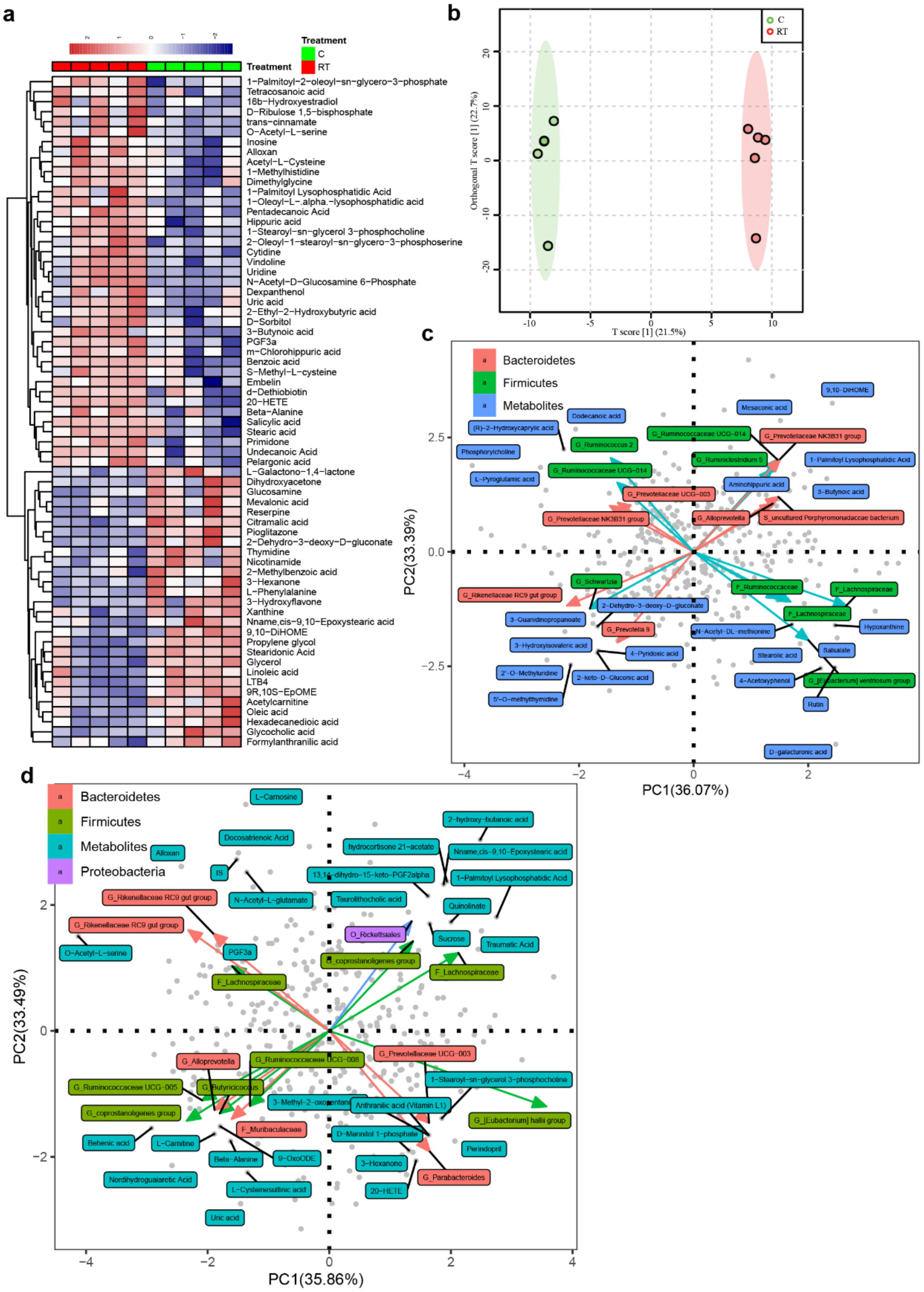

Supplementary Figure 9 Different metabolomic alterations in the negative model in serum and strong correlation with the microbiome of the contents and epithelial surface of the cecum. **a** Heatmap of the differences in serum metabolomic metabolites under

cold stress. **b** Partial least-squares discriminant analysis (PLS-DA) of microbial metabolites in serum. **c** Correlation of the metabolites in serum with the microbiome of the cecal contents. **d** Correlation of the metabolites with the microbiome of the epithelial surface of the cecum.

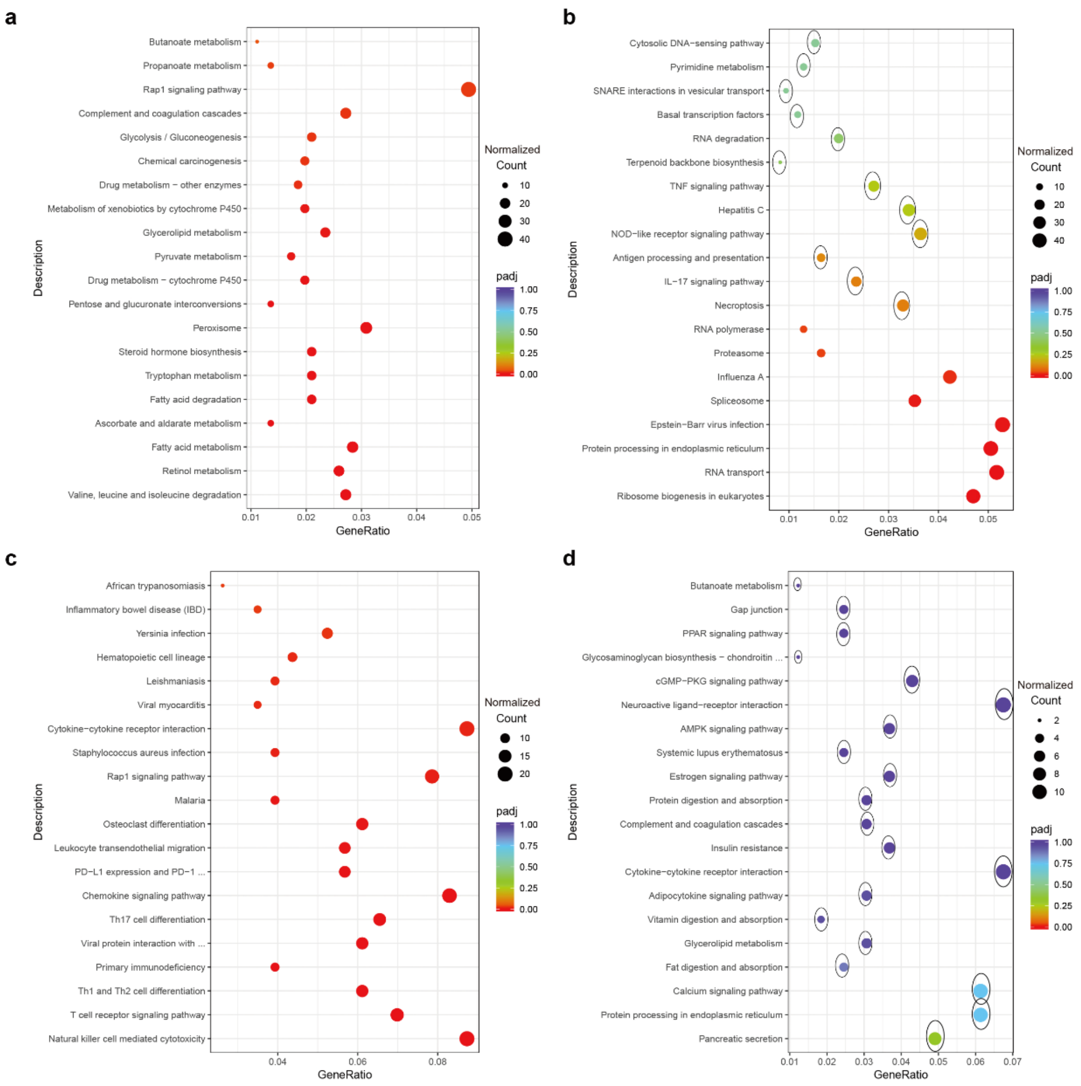

Supplementary Figure 10 Summary of pathways identified at the RNA expression level by RNA seq. **a** Downregulated pathways under cold stress compared with RT in the liver of piglets. **b** Upregulated pathways under cold stress compared with RT in the liver of piglets. **c** Downregulated pathways under cold tress compared with RT in the fat of piglets. **d** Upregulated pathways under cold stress compared with RT in the fat of piglets. The circled nodes indicate results that are not significant (padj > 0.05), and unmarked nodes show significant findings (padj <0.05).

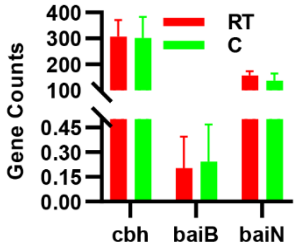

Supplementary Figure 11 The counts of genes involved in secondary bile acid biosynthesis in the gut microbiota of the cecum content by metagenomic sequencing. Cbh, conjugated bile acid hydrolase, baiB, bile acid-coenzyme A ligase, baiN, 3-dehydro-bile acid delta (4,6)-reductase.

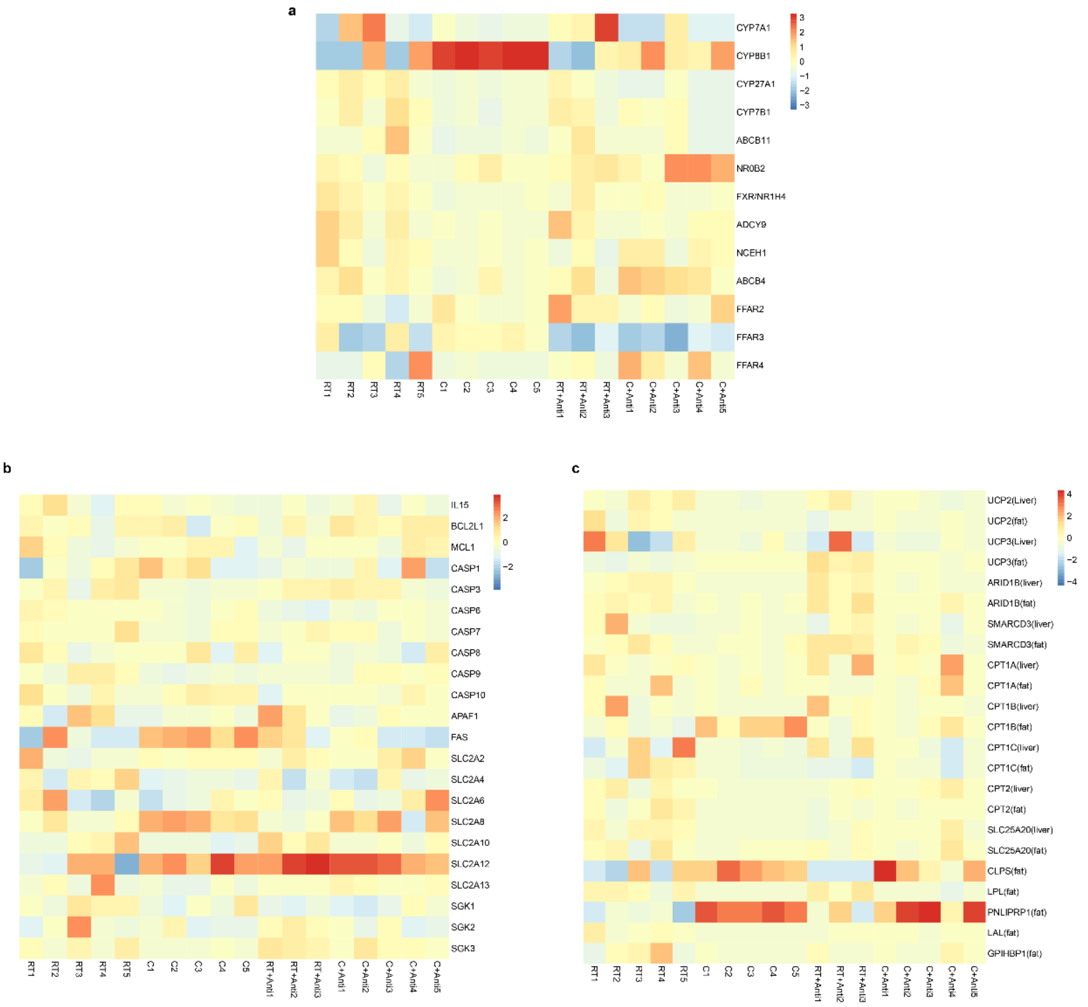

Supplementary Figure 12 Heatmaps showing the variability between samples, (**a, b, c**) related to Figure 6.
