# Supplementary material for: Porcine gut microbiota in mediating host metabolic adaptation to cold stress
Source: NPJ Biofilms Microbiomes. 2022 Apr 5;8:18. doi: 10.1038/s41522-022-00283-2 (PMC8983680; doi:10.1038/s41522-022-00283-2)
Supplement: Supplementary file 3 — Reporting Summary [file 41522_2022_283_MOESM3_ESM.pdf]

nature portfolio

Corresponding author(s): NPJBIOFILMS-01348R3 Jiandui Mi

Last updated by author(s): Feb 15, 2022

Reporting Summary

Nature Portfolio wishes to improve the reproducibility of the work that we publish. This form provides structure for consistency and transparency in reporting. For further information on Nature Portfolio policies, see our Editorial Policies and the Editorial Policy Checklist.

Statistics

For all statistical analyses, confirm that the following items are present in the figure legend, table legend, main text, or Methods section.

n/a	Confirmed	
☐	☒	The exact sample size (*n*) for each experimental group/condition, given as a discrete number and unit of measurement
☐	☒	A statement on whether measurements were taken from distinct samples or whether the same sample was measured repeatedly
☐	☒	The statistical test(s) used AND whether they are one- or two-sided<br>*Only common tests should be described solely by name; describe more complex techniques in the Methods section.*
☐	☒	A description of all covariates tested
☐	☒	A description of any assumptions or corrections, such as tests of normality and adjustment for multiple comparisons
☐	☒	A full description of the statistical parameters including central tendency (e.g. means) or other basic estimates (e.g. regression coefficient) AND variation (e.g. standard deviation) or associated estimates of uncertainty (e.g. confidence intervals)
☐	☒	For null hypothesis testing, the test statistic (e.g. *F*, *t*, *r*) with confidence intervals, effect sizes, degrees of freedom and *P* value noted<br>*Give P values as exact values whenever suitable.*
☐	☒	For Bayesian analysis, information on the choice of priors and Markov chain Monte Carlo settings
☐	☒	For hierarchical and complex designs, identification of the appropriate level for tests and full reporting of outcomes
☐	☒	Estimates of effect sizes (e.g. Cohen's *d*, Pearson's *r*), indicating how they were calculated

Our web collection on statistics for biologists contains articles on many of the points above.

Software and code

Policy information about availability of computer code

Data collection	The data collection was performed by QIIME2.2019.10, Megahit1.2.9, Prodigal2.6.3, eggNOG-mapper2.0.1, Salmon1.1.0, MetaWRAP1.3.1, GTDB-tk1.5.0,ProteoWizard3, MetaboAnalyst4.0, feature counts and R3.6.3 software. All software are open source. Code and detailed information are available on github (https://github.com/mijiandui/Porcine-gut-microbiota-in-mediating-host-metabolic-adaptation-to-cold-stress).
Data analysis	The data analyses were performed by QIIME2.2019.10, Megahit1.2.9, Prodigal2.6.3, eggNOG-mapper2.0.1, Salmon1.1.0, MetaWRAP1.3.1, GTDB-tk1.5.0,ProteoWizard3, MetaboAnalyst4.0, feature counts and R3.6.3 software. All software are open source. Code and detailed information are available on github (https://github.com/mijiandui/Porcine-gut-microbiota-in-mediating-host-metabolic-adaptation-to-cold-stress).

For manuscripts utilizing custom algorithms or software that are central to the research but not yet described in published literature, software must be made available to editors and reviewers. We strongly encourage code deposition in a community repository (e.g. GitHub). See the Nature Portfolio guidelines for submitting code & software for further information.

Data

Policy information about availability of data

All manuscripts must include a data availability statement. This statement should provide the following information, where applicable:

- Accession codes, unique identifiers, or web links for publicly available datasets
- A description of any restrictions on data availability
- For clinical datasets or third party data, please ensure that the statement adheres to our policy

The raw sequence data are deposited in the ENA Sequence Read Archive under accession PRJEB44118. The list of figures that have associated raw data were upload with raw data. There was no restrictions on the data availability.

Field-specific reporting

Please select the one below that is the best fit for your research. If you are not sure, read the appropriate sections before making your selection.

☒ Life sciences ☐ Behavioural & social sciences ☐ Ecological, evolutionary & environmental sciences

For a reference copy of the document with all sections, see nature.com/documents/nr-reporting-summary-flat.pdf

Life sciences study design

All studies must disclose on these points even when the disclosure is negative.

Sample size	A total of 20 piglets were randomly divided into the room temperature (RT), room temperature with antibiotics (RT+antibiotics), cold stress (C) and cold stress with antibiotics (cold+ antibiotics) groups with 5 replicates per group according to body weight.
Data exclusions	No data were exclude.
Replication	A total of 20 piglets were randomly divided into the room temperature (RT), room temperature with antibiotics (RT+antibiotics), clod stress (C) and clod stress with antibiotics (Cold+ antibiotics) groups with 5 replicates per group according to body weight.
Randomization	The samples were allocated randomly into groups.
Blinding	NA

Reporting for specific materials, systems and methods

We require information from authors about some types of materials, experimental systems and methods used in many studies. Here, indicate whether each material, system or method listed is relevant to your study. If you are not sure if a list item applies to your research, read the appropriate section before selecting a response.

Materials & experimental systems

n/a	Involved in the study	
☒	☐	Antibodies
☒	☐	Eukaryotic cell lines
☒	☐	Palaeontology and archaeology
☐	☒	Animals and other organisms
☒	☐	Human research participants
☒	☐	Clinical data
☒	☐	Dual use research of concern

Methods

n/a	Involved in the study	
☒	☐	ChIP-seq
☒	☐	Flow cytometry
☒	☐	MRI-based neuroimaging

Animals and other organisms

Policy information about studies involving animals; ARRIVE guidelines recommended for reporting animal research

Laboratory animals	Piglets were reared in turning carts, with each turning cart divided into pens. Each turning cart included 3 experimental animals. Piglets were provided with feed and water ad libitum; the remaining feed in the trough was cleaned up with new feed added at 08:00 and 18:00, and the cart and environment were simultaneously cleaned. After one week of adaptation, the RT+antibiotics and cold +antibiotics groups received drinking water containing neomycin (227.30 mg/day), streptomycin (113.60 mg/day), vancomycin (113.60 mg/day), metronidazole (227.30 mg/day), bacitracin (2.27 g/day), ciprofloxacin (284.10 mg/day), ceftazidime (227.30 mg/ day), gentamicin (396.40 mg/day), and penicillin (227.30 mg/day) until the end of the experiment. During the depletion period, 2 piglets in the RT+antibiotics group were removed because of health problems. Piglets in the nonantibiotic group did not receive any

	drugs or antibiotics, and the health status of all piglets was recorded. One week after receiving antibiotics, the cold stress groups were exposed to a low temperature of 18°C for 48 h.
Wild animals	NA
Field-collected samples	We collected samples from 18 piglets, including 5 piglets in the RT group, 5 piglets in the cold stress group, 5 piglets in the cold stress group treated with antibiotics, and 3 piglets in the RT group treated with antibiotics. Blood was collected from the anterior vena cava of each piglet to extract serum for nontargeted metabolomics analysis, determine the composition of bile acid, and measure other parameters. Each intestinal segment was punctured and carefully cut. Cecal contents and epithelial surface samples were collected for 16S rRNA and metagenomic analysis and SCFA measurement. Then, the remaining contents were removed, and the cecal wall was cut and washed with 4°C normal saline. A sterile glass slide was used to gently scrape the intestinal mucosa, and other tissue samples (liver, spleen, and thymus) were first weighed and collected in the same way. All utensils used in the sample collection process were sterilized, and each group of samples was divided into 3 sterile centrifuge tubes, quickly placed in liquid nitrogen, transferred to the laboratory, and stored at -80°C for testing. All samples from each pig were collected within 30 min after slaughter. The DNA samples were stored at -20? for 16S rRNA gene amplicon and metagenomic sequencing.
Ethics oversight	The experimental design and procedures were conducted according to the institutional guidelines for the care and use of experimental procedures involving animals were approved by the Animal Experimental Committee of South China Agricultural University (SYXK2014-0136).

Note that full information on the approval of the study protocol must also be provided in the manuscript.
